# Clustering of lymphoid neoplasms by cell of origin, somatic mutation and drug usage profiles: a multi-trait genome-wide association study

**DOI:** 10.1038/s41408-025-01351-4

**Published:** 2025-08-29

**Authors:** Murat Güler, Federico Canzian

**Affiliations:** 1 Genomic Epidemiology Group, German Cancer Research Center (DKFZ), Heidelberg, Germany; 2 Medizinische Fakultät Heidelberg, Universität Heidelberg, Heidelberg, Germany

**Keywords:** Risk factors, Haematological cancer, Cancer genetics

## Abstract

Lymphoid neoplasms (LNs) are heterogeneous malignancies arising from lymphoid cells, displaying diverse clinical and molecular features. Although LNs are collectively frequent, individual subtypes are rare, posing challenges for genetic association studies. Indeed, genome-wide association studies (GWAS) explained only a fraction of the heritability. Shared genetic susceptibility and overlapping risk factors suggest a partially common etiology across subtypes. We employed a multi-trait GWAS strategy to improve discovery power by leveraging pleiotropy among LN subtypes. We defined LN phenoclusters based on cell of origin, somatic mutation profiles, and approved therapeutic agents. Using data from three large cohorts—the UK Biobank, Million Veteran Program, and FinnGen—we analyzed 31,937 LN cases and 1.2 million controls across 8 individual subtypes and 7 phenoclusters. We replicated the novel associations in two independent cohorts (All of Us and the Prostate, Lung, Colorectal, and Ovarian Cancer Screening Trial) with 2892 LN cases and 165,791 controls. We identified 76 genome-wide significant loci for individual subtypes or subtype clusters, including 20 novel associations. We identified the subtypes contributing to each locus, putative candidate causal variants, and genes underlying the associations, and found enrichment of specific cell types, biological processes, and drugs associated with LN risk genes. Overall, this study identified new LN genetic risk loci and candidate genes, providing insights that may inform novel therapeutic approaches.

## Introduction

Lymphoid neoplasms (LNs) are a diverse group of malignancies arising from lymphoid cells at various stages of differentiation. While individual LN subtypes are rare, together they comprise more than 60 clinically distinct entities and rank among the most common cancers worldwide [1]. Risk factors for LNs include inherited genetic variants, viral infections, environmental exposures, and immune dysregulation [2]. Despite their heterogeneity, shared susceptibility across subtypes has been observed—including familial clustering—suggesting overlapping etiological pathways [3].

Genome-wide association studies (GWASs) identified shared and subtype-specific loci for several LN entities [4–6]. However, these findings explain only a limited portion of heritability. For instance, GWAS heritability estimates range from 15.6% for multiple myeloma (MM) [7] to 34% for chronic lymphocytic leukemia (CLL) [8], falling short of heritability estimates from family and twin studies [9]. This “missing heritability” may reflect undetected additive effects, gene–gene or gene–environment interactions, or shared variants with modest effect sizes across related subtypes [10]. Power analyses suggest that sample sizes of 50,000 to over 1 million cases would be required to explain 80% of GWAS heritability for different cancers [11]—a scale that remains infeasible for most individual LN subtypes.

To address these limitations, multi-trait GWAS methods have been proposed as a powerful alternative [12–14]. These approaches exploit pleiotropy—where a genetic variant influences multiple traits—to increase statistical power by aggregating biologically related phenotypes. In the context of LNs, many subtypes share molecular features, therapeutic agents, and developmental origins, suggesting the potential for pleiotropic risk variants.

We hypothesized that grouping LN subtypes into biologically informed “phenoclusters” could improve the discovery of shared and subtype-specific genetic loci. We included CLL, diffuse large B-cell lymphoma (DLBCL), follicular lymphoma (FL), Hodgkin lymphoma (HL), monoclonal gammopathy of undetermined significance (MGUS), MM, mantle cell lymphoma (MCL), marginal zone lymphoma (MZL), peripheral T-cell lymphoma (PTCL), and lymphoplasmacytic lymphoma/Waldenström macroglobulinemia (LPL-WM). We constructed phenoclusters using hierarchical clustering based on three biological and clinical criteria: cell of origin [15–18], somatic mutation profiles, and approved therapeutic agents. We then applied both hypothesis-driven (phenocluster-based) and hypothesis-free (ASSET) multi-trait GWAS frameworks across large biobank cohorts, analyzing over 31,000 LN cases and 1.2 million controls.

## Methods

**Fig. 1:**
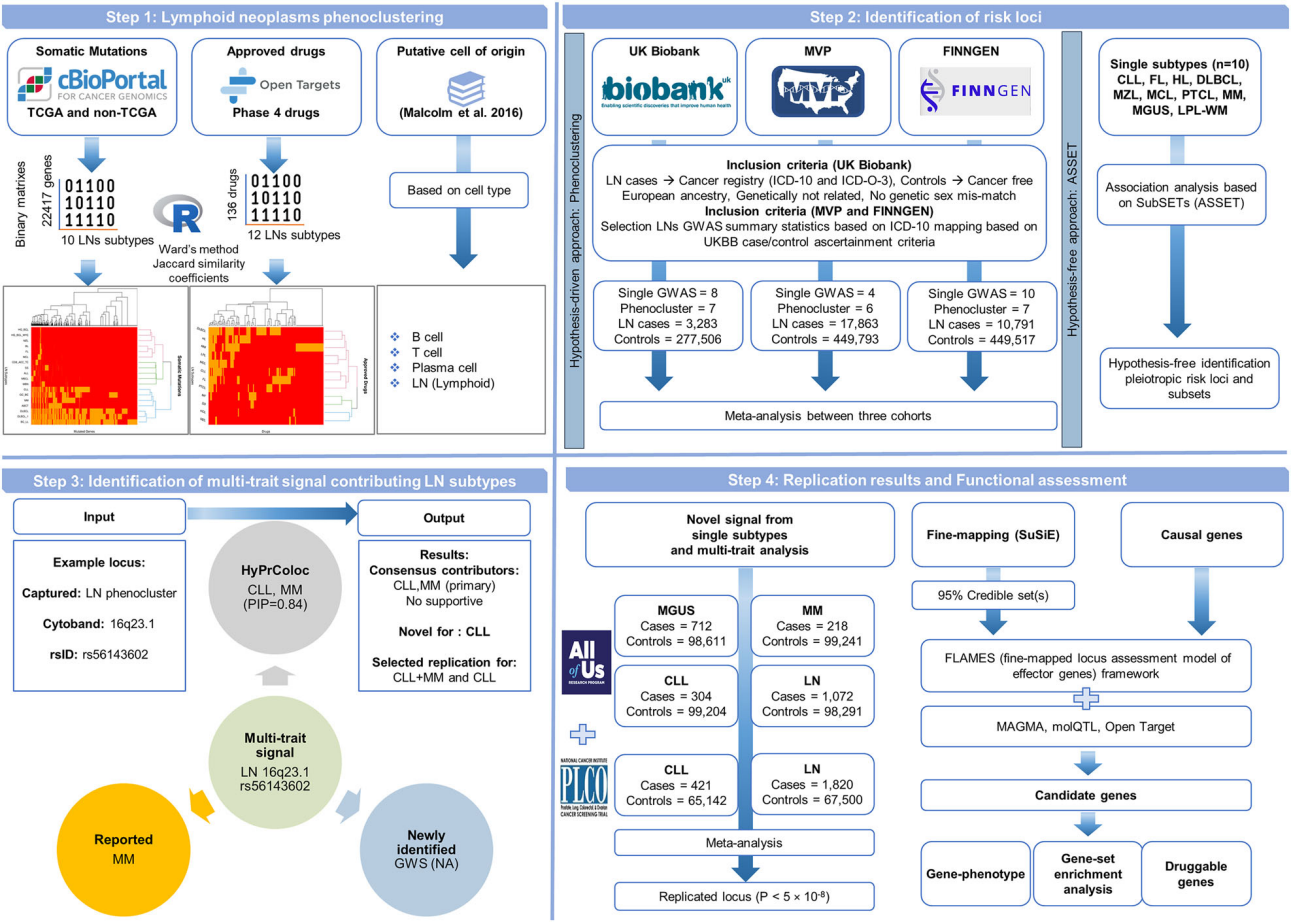
Study design and graphical summary of the methods. A comprehensive pipeline for identifying and characterizing multi-trait genetic signals associated with LN. The pipeline encompasses four organized steps: (1) LN phenoclustering, integrating somatic mutation data (cBioPortal; TCGA and non-TCGA datasets), drug information (Open Targets), and putative cell-of-origin annotations based on cell types, employing Ward’s clustering method to define subtype relationships; (2) Identification of genetic risk loci leveraging large biobank cohorts (UKB, MVP, and FINNGEN), through both hypothesis-free association analysis (SubSETs/ASSET approach) and hypothesis-driven phenocluster-informed analysis for identification of subtype-specific and pleiotropic loci across LN subtypes; (3) Characterization of multi-trait signals, illustrated with an example locus (LN phenocluster, 16q23.1, rs56143602), involving multi-trait colocalization (HyPrColoc), reported and newly identified association for subtypes, and identification of consensus contributor subtypes (CLL, MM primary); and (4) Replication and functional assessment, incorporating independent cohort meta-analysis (PLCO and AoU), fine-mapping (SuSiE), candidate gene prioritization using FLAMES (Fine-mapped Locus Assessment Model of Effector genes), functional annotation (FUMA: MAGMA, molQTL, Open Target), and detailed gene-based enrichment analyses to reveal LN biology and druggable genes.

An overview of the study design is presented in Fig. 1, with detailed descriptions of each analytical step provided in the Supplementary Information. The complete computational pipeline, including scripts and workflows used for replication of all analyses and figures, is publicly available at https://github.com/biomguler/LN_Phenocluster/.

### Construction of LN phenoclusters using hierarchical clustering

To group LN subtypes based on shared biological and clinical features, we performed hierarchical clustering using three modalities:

1. Cell-of-origin data were curated from the published literature [15–18], assigning each LN subtype to a major developmental lineage (i.e., B cell, plasma cell, and T cell).
2. Somatic mutation profiles were obtained from the cBioPortal database and converted into a binary matrix representing the presence or absence of mutations in 22,417 genes for each subtype (Supplementary Data 1).
3. Drug usage profiles were derived from the Open Targets Platform and transformed into binary matrices to indicate whether a given drug was approved for each LN subtype (Supplementary Data 2).

Each clustering was performed using Ward’s minimum variance method and Jaccard similarity coefficient, appropriate for binary data. Phenocluster definitions were guided by both algorithmic structure and biological interpretability (Supplementary Figs. 1–4).

An additional phenocluster including all LN subtypes was created to account for their shared hematopoietic origin.

### Study populations and association testing

We conducted GWASs for individual LN subtypes and derived phenoclusters using three large, population-based cohorts: the UK Biobank (UKB), the Million Veteran Program (MVP), and FinnGen. Cohort description, data acquisition, and selection of cases and controls are extensively described in the Supplementary Information and Supplementary Tables 1–3. Summary statistics from each cohort were meta-analyzed using inverse-variance weighted fixed-effects models implemented in METAL [19]. We focused on only European ancestry due to a lack of statistical power for other ancestries.

### Phenotype selection and association testing

We selected eight individual LN subtypes and seven phenoclusters for association analysis, retaining only phenotypes with ≥100 cases to minimize bias from case imbalance. In UKB, association testing was performed using REGENIE v3.2 [20], adjusting for age (at diagnosis for cases; at recruitment for controls), sex, genotyping array, and the first ten principal components (UKB Data-Field 22009) [21].

For FinnGen and MVP, phenocluster-level summary statistics were constructed by meta-analyzing available subtype-level results. Exceptions included the broad LN phenotype and MM-MGUS phenocluster, for which full summary statistics were directly available (Supplementary Table 4).

### Association analysis based on subsets (ASSET)

To complement the phenocluster-based approach in a hypothesis-free manner, we employed ASSET [14]. ASSET is a subset-based meta-analysis framework that systematically evaluates all possible combinations of traits to detect association signals, accounting for heterogeneity in genetic effects.

We performed both one-sided and two-sided ASSET analyses across ten LN subtypes: CLL, DLBCL, FL, HL, MGUS, MM, MCL, MZL, PTC, and LPL-WM. Subtypes were included if individual GWAS summary statistics were available from at least one cohort, with MCL and PTCL included based on data from FinnGen only.

One-sided ASSET was used to identify subsets of subtypes that showed associations in the same direction, either risk-increasing or risk-decreasing. Two-sided ASSET allowed for directional heterogeneity, enabling the detection of loci with opposite effects across subtypes by combining association signals using a chi-squared test.

### Testing global genetic correlation

To quantify the shared genetic architecture among LN subtypes and phenoclusters, we estimated genome-wide genetic correlations using linkage disequilibrium score regression (LDSC), implemented with the LDSC v1.0.1 software [22]. Summary statistics from genome-wide association analyses of individual LN subtypes and phenoclusters were processed using the munge_sumstats.py utility provided in the LDSC package. Analyses were restricted to HapMap3 variants, following recommended best practices to ensure reliability of heritability and correlation estimates.

Variants with a minor allele frequency (MAF) below 5% were excluded from the analysis. In addition, we removed variants located within the extended major histocompatibility complex (MHC) region on chromosome 6 (25–35 Mb), due to the complex linkage disequilibrium patterns that can bias correlation estimates in this region. Bivariate genetic correlations were calculated between each pair of traits, and statistical significance was determined using a Bonferroni-corrected threshold of *P* ≤ 0.005, accounting for ten unique subtypes tested.

### Definition of independent loci and genomic regions

To define independent genome-wide significant (GWS) loci, we applied the clumping procedure implemented in PLINK [23] using a *P*-value threshold of 5 × 10^−8^, an *R*² threshold of 0.01, and a physical distance window of 1 megabase (Mb) around the index variant (command: -- clump -p1 5e-8 --clump-p2 5e-8 --clump-r2 0.01 --clump-kb 10000) and merged those loci with lead SNPs within 1 Mb of each other to obtain the final independently significant loci. For analyses involving individual LN subtypes, novel loci were defined as those not previously reported for the same subtype. Specifically, a locus was considered novel if its lead single-nucleotide polymorphism (SNP) was located outside a ±1 Mb window from any known lead variant and exhibited low LD (pairwise *R*² < 0.01) with previously reported associations, as detailed in Supplementary Table 5.

### Identification of driver-subtypes and pleiotropic loci

To identify the specific LN subtypes contributing to multi-trait association signals and to classify pleiotropic loci, we used a three-step integrative strategy combining phenocluster-based and subset-based findings. First, we applied Hypothesis Prioritization in Multi-Trait Colocalization (HyPrColoc), a Bayesian framework that detects colocalized association signals and infers likely causal variants shared across traits. HyPrColoc groups traits based on shared regional association patterns and computes a posterior probability (PP) of colocalization for each cluster [24]. Analyses were conducted using default parameters, with prior.1 set to 1 × 10^−^^4^ and prior.c to 0.02, and with the branch-and-bound search algorithm enabled. Subtypes with regional PP values greater than 0.7 were designated as “primary contributors”, while those with lower support were labeled as “supportive contributors”.

Second, we examined subtype-specific GWAS results at each multi-trait locus. Subtypes were classified as primary contributors if they reached genome-wide significance (*P* < 5 × 10^−^^8^) and as supportive contributors if they showed suggestive significance (5 × 10^−^^8^ < *P* < 1 × 10^−^^6^). These annotations were based on results reported in Supplementary Table 6.

Third, we cross-referenced all identified loci with previously reported subtype-specific risk loci. If a subtype exhibited suggestive significance at a locus and had been previously implicated in association with a lead SNP located within ±500 kb and in LD (*R*² ≥ 0.01), it was also considered a primary contributor

By merging primary, supportive, and previously reported contributors, we generated a final list of associated subtypes for each locus. Loci were categorized as pleiotropic if two or more primary subtypes were implicated, as non-pleiotropic if only one primary contributor was identified, and as “potentially pleiotropic” if no clear primary or supportive contributor could be assigned. This classification allowed us to dissect the subtype-specific vs shared genetic basis underlying multi-trait associations.

### Replication of novel loci

We attempted replication of novel associations using summary statistics from two independent cohorts: the All of Us (AoU) Research Program [25] and the Prostate, Lung, Colorectal, and Ovarian (PLCO) Cancer Screening Trial [26]. For individual LN subtypes, replication was limited to CLL, MM and MGUS, for which data were available with sufficient statistical power in AoU and/or PLCO. Supplementary Table 4 shows LN phenotype definitions and case-control numbers.

For loci identified through phenocluster- or ASSET-based analyses, we selected subtypes for replication based on their contributor status. If the novel signal involved one or two contributing subtypes, replication was performed using single-subtype data or a subtype-specific meta-analysis within the replication cohort. If a locus involved three or more contributing subtypes, or if no specific contributors could be confidently assigned, replication was conducted using a broad LN phenotype, defined in AoU and PLCO as a composite of all available LN subtypes.

Meta-analysis of discovery and replication data was performed using inverse-variance weighted fixed-effects models implemented in METAL [19].

A locus was considered replicated if the effect direction was concordant with the discovery analysis, the effect size was of similar magnitude, and the combined meta-analysis reached genome-wide significance.

### Statistical fine-mapping

To identify putative causal variants within associated loci, we performed statistical fine-mapping using the SuSiE (Sum of Single Effects) method [27] with both individual subtypes and phenocluster-level GWAS results. For each GWS locus, we defined a ±500-kilobase (kb) region around the lead SNP as the input window. LD reference matrices were generated using genotype data from 337,491 unrelated British participants of European ancestry in the UKB [28], ensuring population-matched LD structure for accurate posterior inference.

We used the susieR package (version 0.12.35) in R with default parameters. We required 95% credible sets to achieve a posterior inclusion probability (PIP) coverage of at least 0.95, with a minimum pairwise LD threshold of *R*² ≥ 0.5 to ensure variant correlation within sets. Loci located within the extended MHC region were excluded from fine-mapping. Seven of the fine-mapped loci did not yield credible sets and were excluded from downstream interpretations.

### Functional annotation of variants

To explore the molecular mechanisms underlying the identified association signals, we performed functional annotation of fine-mapped variants using the Ensembl Variant Effect Predictor (VEP, version 113) [29]. This included annotation of variant consequences, predicted functional effects, and overlap with known regulatory elements.

We also integrated cis-molecular quantitative trait locus (molQTL) data to assess the regulatory activity of credible set variants. These included expression (eQTLs), splicing (sQTLs), protein (pQTLs), transcript usage (tuQTLs), and single-cell expression QTLs (sceQTLs), sourced from multiple large-scale databases including the eQTL Catalog [30], Open Targets Platform [31], eQTLGen Consortium [32], and the UKB Pharma Proteomics Project [33]. We focused on QTLs derived from hematopoietic tissues and whole blood.

### Locus to gene mapping

To prioritize candidate effector genes at associated risk loci, we applied a multi-pronged locus-to-gene mapping strategy integrating statistical, functional, and regulatory evidence. First, we used FLAMES (Fine-mapped Locus Assessment Model of Effector Genes), a machine learning-based framework that aggregates diverse genomic annotations to predict the most likely effector gene per locus [34].

Second, we conducted gene-based association testing using MAGMA [35], as implemented in FUMA (version 1.5.2) [36]. MAGMA integrates GWAS summary statistics and gene location to compute a gene-level test statistic. Genome-wide significance for MAGMA analyses was defined at a Bonferroni-corrected threshold of *P* = 2.63 × 10^−^^6^, corresponding to 19,010 tested genes.

Third, we used the Open Targets Locus2Gene scoring framework [37] to identify the most likely gene(s) at each locus based on proximity, functional consequence, and regulatory evidence from fine-mapped variants.

Fourth, we integrated cis-molecular QTL annotations from the previously described molQTL databases. Genes were considered supported if they were significantly regulated by variants within the 95% credible set.

Each gene was given a score of 1 if it was prioritized by a given method and of 0 if not. Scores across methods were averaged to generate a composite prioritization score per gene. Genes with support from multiple independent lines of evidence were flagged as high-confidence candidates for functional follow-up.

### Enrichment analysis identified genes and drug targets

To investigate the biological relevance and translational potential of the prioritized genes, we conducted a series of enrichment analyses focusing on tissue specificity, functional pathways, and therapeutic targeting. We first performed tissue- and cell-type-specific enrichment analysis using the Web-based Cell-type Specific Enrichment Analysis (WebCSEA) tool [38]. This tool evaluates gene expression patterns across 1355 human tissues and cell types and provides both nominal and permutation-based *P*-values for enrichment. Analyses were performed separately for the full set of prioritized genes, as well as the subset derived exclusively from novel loci.

To explore functional protein–protein interactions (PPIs), we queried the STRING database (version 12) [39]. Enrichment for Gene Ontology (GO) biological processes was assessed using STRING’s built-in annotation framework. Terms were considered significantly enriched if they met a false discovery rate (FDR) threshold of <0.05, and a minimum of two genes in the enrichment set was required to prevent false enrichment signals.

To assess therapeutic relevance, we investigated drug–gene interactions (DGI) using the Drug–Gene Interaction Database (DGIdb) [40]. Identified gene–drug pairs were annotated with Anatomical Therapeutic Chemical (ATC) codes from DrugBank. We then tested for enrichment of ATC first- and second-level categories using Fisher’s exact test, with significance defined at FDR < 0.05 relative to the full set of ATC annotations in DrugBank.

In parallel, we queried Open Targets for known drug interactions involving our prioritized genes, focusing on agents with approved or investigational indications based on ChEMBL annotations [41]. For each gene–drug pair, we manually obtained data on clinical status and indication using DrugBank and ClinicalTrials.gov to determine relevance to LN. Genes located in the MHC region were excluded from all enrichment and interaction analyses.

## Results

### Hierarchical clustering of LNs

We performed hierarchical clustering using three independent criteria. Cell-of-origin-based clustering grouped LN subtypes into three major categories: B cell–derived neoplasms (Cell-B), plasma cell–derived neoplasms (Cell-P), and T cell–derived neoplasms (Cell-T). The specific subtype composition of each group is provided in Supplementary Table 4. Due to the limited sample size of the Cell-T group and the small number of cases per individual subtype (PTCL = 49, MF = 65, SS = 2), Cell-T was excluded from downstream analyses.

Somatic mutation-based clustering was performed using binary profiles of 22,417 somatically mutated genes in LN subtypes. This yielded three clusters: Soma-G1, Soma-G2, and Soma-G3, which was excluded due to low sample size. The composition of Soma-G1 and Soma-G2 clusters is detailed in Supplementary Fig. 3.

Drug-based clustering, using shared approved treatment profiles, identified three groups. However, only Drug-G1 was retained for further analysis. The other two groups—Drug-G2 (comprising MF and SS) and Drug-G3 (comprising HCL and MZL)—were excluded due to small sample sizes (Supplementary Fig. 4). The extended results for the phenoclusters are given in the Supplementary Information.

### GWAS of individual LN subtypes and phenoclusters

Following subtype selection and phenocluster construction across the discovery cohorts, we performed genome-wide meta-analyses for eight individual LN subtypes and seven phenoclusters, using a shared control group (Supplementary Table 4).

In parallel, we conducted subset-based association testing (ASSET) to identify pleiotropic loci across ten LN subtypes: eight available across cohorts and two additional subtypes—MCL and PTCL—which were analyzed using FinnGen data only.

Genomic inflation was not observed for any phenotype. All test statistics were well-calibrated, with genomic inflation factors λ_gc_ ≤ 1.1 (Supplementary Table 7), indicating no substantial population stratification or systematic bias across analyses.

### Identified risk loci from individual LN subtypes meta-analysis

**Fig. 2:**
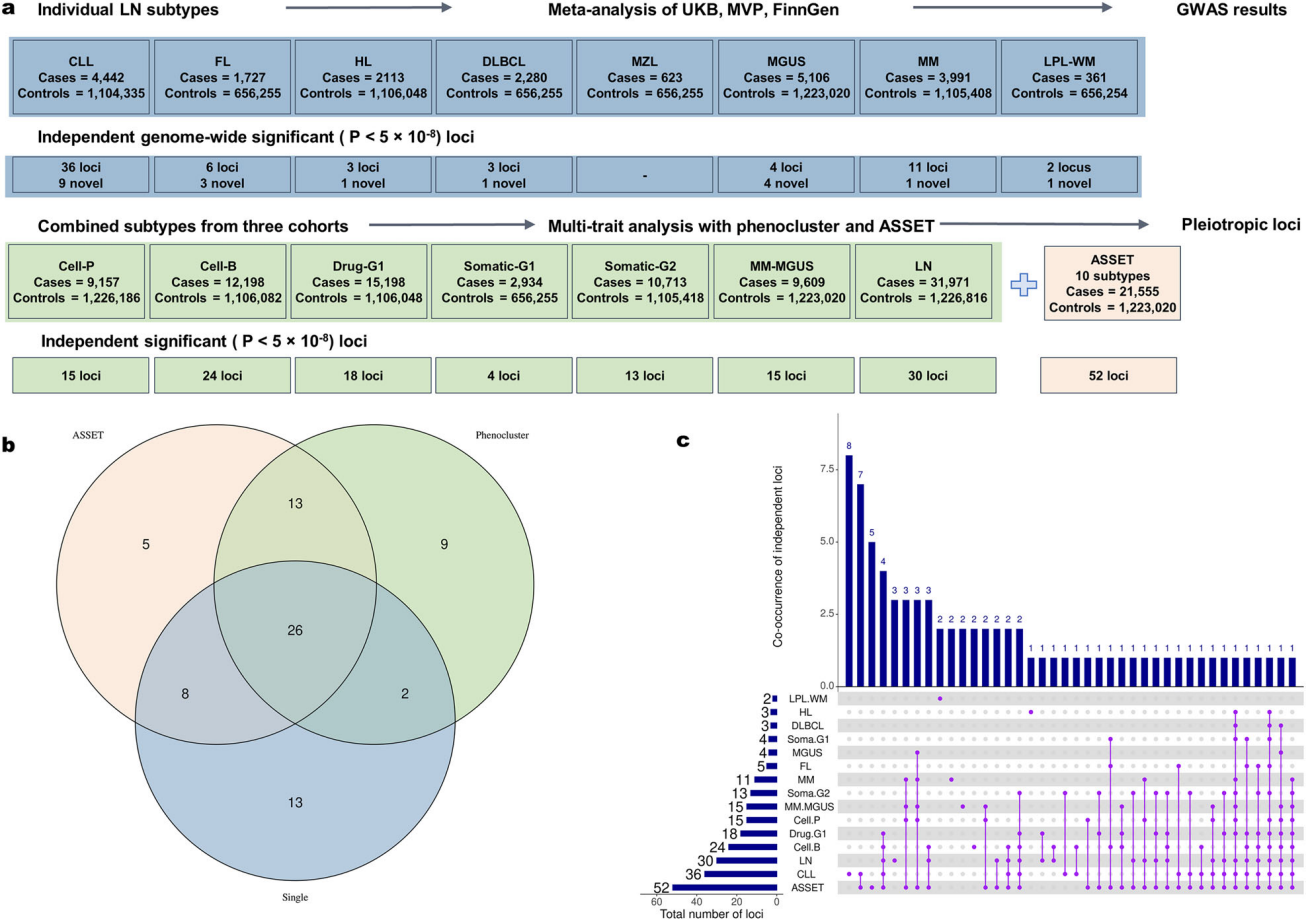
The single subtype and phenocluster analyses reveal 76 unique LN risk loci. **a** Summaries of the individual LN subtypes and multi-trait (phenocluster and ASSET) approach sample sizes, and the detected number of GWS loci. Eight LNs subtypes were meta-analyzed between the three discovery cohorts, and we identified 49 subtype-specific independent signals. We used jointly analyzed data to create 7 phenoclusters, and the hypothesis-free ASSET approach was employed to identify all possible pleiotropic signals. This captured 63 multi-trait signals. **b** Venn diagram of the overlap between 76 unique significant loci across the single-subtype (blue), phenocluster (green), and ASSET (orange) GWAS. **c** UpSet plot illustrating the overlap among 76 unique, significant, and independent genetic signals identified in single-subtype and multi-trait analyses of LNs. Each vertical bar represents the number of signals corresponding to a specific phenotype or combination of phenotypes. The connected purple lines and dots below the bars indicate the co-occurrence of signals across those phenotypes or analysis types. For example, the first vertical bar corresponds to 8 loci uniquely associated with CLL, not shared with other subtypes and not captured by any multi-trait method. The second bar represents seven CLL loci also identified by ASSET, indicating shared signals between single-subtype and multi-trait analyses.

Genome-wide meta-analyses for eight individual LN subtypes across the discovery cohorts yielded a total of 49 independent GWS loci (*P* < 5 × 10^−8^) totaling 65 associations (some loci have been counted multiple times if they are GWS for more than one subtype), of which 20 represented novel associations not previously reported for the corresponding subtype (Supplementary Table 6 and Fig. 2b, blue circle).

To validate our novel findings, we conducted replication analyses using independent datasets from the PLCO and AoU cohorts where data were available. Replication results for the novel loci are summarized in Supplementary Table 6 and presented in Table 1. Among the nine novel CLL loci, eight achieved genome-wide significance in the combined meta-analysis, with the strongest replication observed for 10q22.1 (rs142239370, *PRF1*) and 10q23.33 (rs11187157, *HHEX*). The locus at 10q23.33 has previously been reported as novel and pleiotropic for non-HL subtypes [4], while our analysis revealed it as specific for CLL. Several additional CLL loci, including 3q28 (*LPP*) and 12q24.22 (*MAP1LC3B2*), showed consistent effects and replicated with low heterogeneity. For MGUS, all four novel loci replicated with consistent direction of effect, including 6p25.3 (rs115116856, *EXOC2*/*IRF4*), a rare variant with a strong effect (OR = 2.14, *P* = 1.76 × 10^−13^). The protective variant at 2p23.3 (rs7577599, *DTNB*) also replicated with high confidence. The novel MM locus at 1q23.1 (rs56179914, *CD5L*/*FCRL3*) was directionally consistent in the replication cohort, though statistical power was limited.

**Table 1:** Novel risk loci identified for individual LN subtypes.

LN subtype	Variant						Discovery (UKBB + MVP + FINNGEN)			Replication			Combined		
	Cytoband	rsID	Reference	Alternative	AF	Gene(s)^a^	OR (95%CI)	*P*	*P*_het_	Cohort	OR (95%CI)	*P*	OR (95%CI)	*P*	*P*_het_
CLL	1p34.2	rs873917	T	G	0.687	*NT5C1A*	1.14 (1.09–1.20)	3.15E-08	0.66	PLCO+AoU	1.04 (0.93–1.16)	4.97E-01	1.13 (1.08–1.18)	7.69E-08	0.25
**CLL**	**3p24.1**	**rs388368**	**T**	**C**	**0.471**	***EOMES***	**1.13 (1.09–1.18)**	**1.23E-08**	**0.06**	**PLCO** + **AoU**	**1.08 (0.97–1.20)**	**1.40E-01**	**1.13 (1.08–1.17)**	**5.72E-09**	**0.73**
**CLL**	**3q28**	**rs1849913**	**G**	**A**	**0.729**	***LPP***	**1.17 (1.11–1.23)**	**1.17E-09**	**0.16**	**PLCO** + **AoU**	**1.12 (0.99–1.26)**	**6.63E-02**	**1.16 (1.11–1.21)**	**2.69E-10**	**0.72**
**CLL**	**6p22.3**	**rs72098212**	**AGTT**	**A**	**0.034**	***JARID2***	**1.39 (1.24–1.55)**	**4.40E-09**	**0.14**	**PLCO** + **AoU**	**0.98 (0.71–1.35)**	**9.09E-01**	**1.34 (1.21–1.48)**	**3.46E-08**	0.04^*^
**CLL**	**10q22.1**	**rs142239370**	**A**	**C**	**0.040**	***PRF1***	**1.52 (1.38–1.68)**	**1.07E-16**	**0.22**	**PLCO** + **AoU**	**1.57 (1.21–2.04)**	**5.95E-04**	**1.53 (1.39–1.67)**	**2.88E-19**	**0.50**
**CLL**	**10q23.33**	**rs11187157**	**T**	**C**	**0.440**	***HHEX***	**1.19 (1.14–1.24)**	**5.88E-15**	**0.09**	**PLCO** + **AoU**	**1.25 (1.12–1.40)**	**4.21E-05**	**1.20 (1.15–1.24)**	**1.74E-18**	**0.52**
**CLL**	**12q24.22**	**rs7133288**	**G**	**T**	**0.259**	***MAP1LC3B2***	**1.15 (1.09–1.20)**	**2.66E-08**	**0.21**	**PLCO** + **AoU**	**1.08 (0.96–1.22)**	**1.87E-01**	**1.14 (1.09–1.19)**	**1.50E-08**	**0.47**
**CLL**	**13q14.3**	**rs6561593**	**G**	**A**	**0.061**	***DLEU7***	**1.27 (1.17–1.38)**	**4.48E-09**	**0.30**	**PLCO** + **AoU**	**1.07 (0.87–1.33)**	**5.11E-01**	**1.25 (1.16–1.34)**	**1.06E-08**	**0.35**
**CLL**	**19p13.3**	**rs12971302**	**T**	**C**	**0.322**	***CD70***	**1.14 (1.09–1.19)**	**2.92E-08**	**0.40**	**PLCO** + **AoU**	**1.06 (0.95–1.19)**	**3.02E-01**	**1.13 (1.08–1.18)**	**3.47E-08**	**0.50**
**MGUS**	**2p23.3**	**rs7577599**	**T**	**C**	**0.189**	***DTNB***	**0.84 (0.80–0.89)**	**5.71E-10**	**0.18**	**AoU**	**0.84 (0.74–0.97)**	**1.41E-02**	**0.84 (0.80–0.88)**	**5.57E-12**	**1.00**
**MGUS**	**3p22.1**	**rs6781529**	**C**	**T**	**0.164**	***ULK4***	**1.19 (1.14–1.25)**	**3.08E-12**	**0.10**	**AoU**	**1.26 (1.09–1.45)**	**1.20E-03**	**1.20 (1.15–1.25)**	**1.06E-15**	**0.45**
**MGUS**	**6p25.3**	**rs115116856**	**T**	**C**	**0.008**	***EXOC2, IRF4***	**2.09 (1.69–2.59)**	**1.28E-11**	**0.40**	**AoU**	**2.67 (1.40–5.07)**	**1.96E-03**	**2.14 (1.75–2.62)**	**1.76E-13**	**0.48**
**MGUS**	**17p11.2**	**rs4273077**	**A**	**G**	**0.094**	***TNFRSF13B***	**1.25 (1.17–1.32)**	**1.23E-12**	**0.23**	**AoU**	**1.07 (0.90–1.28)**	**4.29E-01**	**1.23 (1.16–1.30)**	**1.21E-12**	**0.10**
MM	**1q23.1**	**rs56179914**	**C**	**A**	**0.041**	***CD5L, FCRL3***	**1.44 (1.27–1.64)**	**3.64E-08**	**0.87**	**AoU**	**1.35 (0.83–2.21)**	**2.30E-01**	**1.43 (1.27–1.62)**	**1.11E-08**	**0.80**
DLBCL	6p22.2	rs71557353	T	G	0.150	*ZNF184, ZNF322*	1.28 (1.17–1.39)	2.54E-08	0.47						
FL	3p24.1	rs12497690	A	C	0.372	*EOMES*	1.25 (1.17–1.34)	1.71E-10	0.48						
FL	6p22.2	rs2690093	G	A	0.166	*CARMIL1*	1.29 (1.19–1.39)	9.03E-10	0.72						
FL	10q22.1	rs142239370	A	C	0.040	*PRF1*	1.60 (1.36–1.89)	1.08E-08	0.96						
HL	6q16.1	rs146965926	A	G	0.002		4.21 (2.52–7.02)	3.72E-08	0.90						
LPL-WM	4p15.32	rs550571596	T	A	0.003	*FAM184B, NCAPG*	11.88 (5.09–27.77)	1.10E-08	0.55						

Lead variants identified after individual LN subtypes meta-analysis, which have not been previously reported. GWS loci after combining discovery and replication are reported in bold. The DLBCL, FL, HL, and LPL-WM subtypes' putative novel loci were not available for replication in the PLCO and AoU cohorts. *AF* alternative (effect) allele frequency, *OR* odds ratio, 95% *CI* 95% confidence interval, Phet P-value for meta-analysis heterogeneity.

^a^The mapped genes were identified with a combination of various methods (see Methods, Locus to gene mapping section).

^*^P_het_ < 0.05.

Replication could not be pursued for newly identified loci in FL, DLBCL, HL, and LPL-WM due to the absence of suitable subtype-specific data in external cohorts. Nonetheless, all GWS loci from the discovery meta-analyses were subsequently incorporated into the subsequent multi-trait analyses.

### Multi-trait approach-based identified risk loci and genetic overlap

To uncover shared genetic susceptibility across LNs, we applied both a hypothesis-driven phenocluster framework and a hypothesis-free subset-based approach (ASSET). These complementary multi-trait strategies identified 63 independent GWS associated with LN risk (Supplementary Tables 8–9 and Fig. 2b, union of the orange and green circles).

**Fig. 3:**
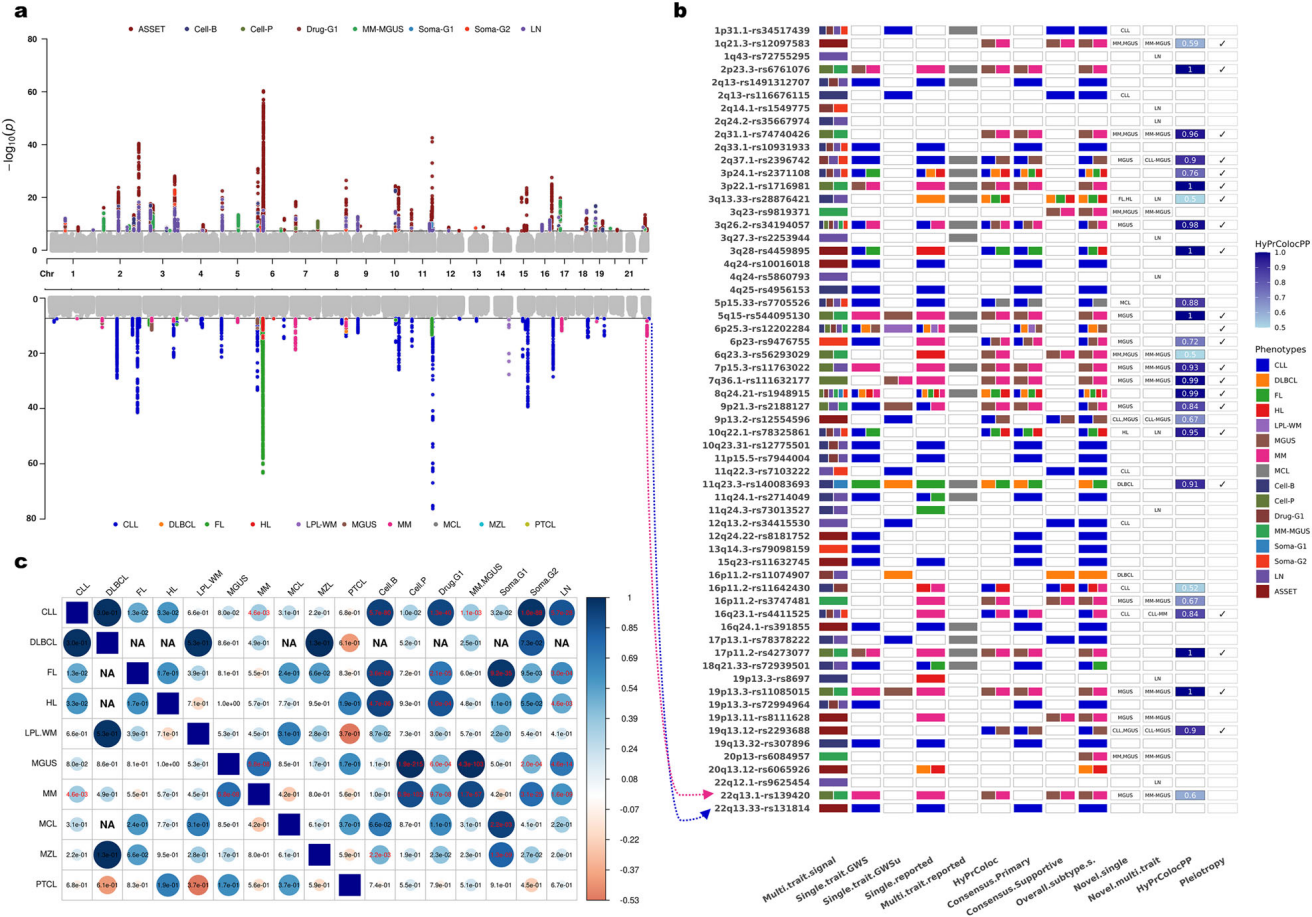
Identified multi-trait signals and individual LN subtypes. **a** The Miami plot shows signals captured in multi-trait (up) and individual subtype GWASs (down). **b** Summary result of pleiotropy assessment and identified contributor subtypes for the multi-trait signals. Each phenocluster and subtype is shown with distinct colors. The arrows, as an example, link two multi-trait signals on chromosome 22 and their contributors with individual subtype and multi-trait associations. **c** The corrplot shows bivariate genetic correlation between subtypes and phenoclusters. The size and color of the circles show the direction and magnitude of the correlation, while *P*-values are shown inside. Significant results after multiple test correction are given in red color.

To delineate the subtype contributions underlying these signals, we integrated evidence from single-subtype GWAS (Supplementary Table 6), multi-trait colocalization using HyPrColoc (Supplementary Table 10), and previously reported LN-associated loci (Supplementary Table 5). Through this framework, primary and supportive contributing subtypes were defined for 55 of the 63 loci (Supplementary Table 11). Of these, 19 loci were primarily driven by CLL, including five previously identified multi-trait loci. Twenty-six loci demonstrated pleiotropy, with contributions from at least two subtypes. Seventeen loci had no clearly assignable contributor subtype, including nine with supportive subtype evidence and eight with no identifiable subtype, while one locus was specific to DLBCL (16p11.2). (Supplementary Tables 11–12). The distribution of contributing subtypes is shown in Fig. 3a, b, highlighting both subtype-specific and pleiotropic patterns of genetic risk across the LN spectrum.

Replication analyses were performed for novel loci with defined contributors, using corresponding single-subtype data. For signals involving multiple contributing subtypes (e.g., MM and CLL), we conducted subtype-specific and/or combined meta-analyses. For loci with undefined or ≥3 contributors—or where novelty was observed in any contributing subtype—replication was conducted using the broad LN phenotype in the PLCO and the AoU. Results are summarized in Supplementary Table 13, with replicated loci presented in Table 2.

**Table 2:** Novel risk loci identified in multi-trait analyses.

Phenotype	Captured by multi-trait(s)	Variant						Discovery (UKB-MVP-FINNGEN)		Replication			Combined		
		Cytoband	rsID	Reference	Alternative	AF	Gene(s)^a^	OR (95% CI)	*P*	Cohort	OR (95% CI)	*P*	OR (95% CI)	*P*	*P*_het_
CLL	LN, Cell-B, Drug-G1, Soma-G2	1p31.1	rs34517439	C	A	0.092	*DNAJB4*	1.18 (1.11–1.27)	8.01E-07	PLCO+AoU	1.24 (1.04–1.46)	1.39E-02	1.19 (1.12–1.27)	3.91E-08	0.07
CLL	Soma-G2, Drug-G1, LN	16q23.1	rs12933037	G	A	0.385	*RFWD3*	0.89 (0.86–0.94)	9.78E-07	PLCO+AoU	0.83 (0.75–0.93)	7.81E-04	0.88 (0.85–0.92)	6.19E-09	0.29
MGUS	Cell-P, MM-MGUS, LN, Soma-G2, Drug-G1, Cell-B	3q26.2	rs34194057	G	T	0.257	*MYNN*	0.90 (0.86–0.94)	4.63E-06	AoU	0.92 (0.82–1.04)	1.89E-01	0.90 (0.87–0.93)	1.73E-08	0.66
MGUS	MM-MGUS, Cell-P	5q15	rs544095130	AT	A	0.295	*ELL2*	0.89 (0.85–0.93)	6.16E-07	AoU	0.97 (0.86–1.09)	5.98E-01	0.89 (0.86–0.93)	4.74E-09	0.16
MGUS	Cell-P, MM-MGUS	6q23.3	rs1179411747	CT	C	0.309	*HBS1L, MYB*	1.11 (1.06–1.16)	4.93E-06	AoU	1.09 (0.96–1.23)	1.70E-01	1.11 (1.07–1.16)	1.57E-08	0.69
MGUS	Cell-P, MM-MGUS, LN	9p21.3	rs2811711	T	C	0.153	*CDKN2A, DMRTA1*	0.85 (0.80–0.91)	7.05E-07	AoU	0.81 (0.70–0.94)	6.62E-03	0.85 (0.80–0.89)	2.60E-09	0.55
MM-MGUS	ASSET	1q21.3	rs12097583	T	C	0.073	*INTS3*	1.20 (1.13–1.28)	3.91E-09	AoU	1.03 (0.86–1.25)	7.25E-01	1.18 (1.12–1.26)	1.13E-08	0.14
MM-MGUS	MM-MGUS, Cell-P	2q31.1	rs74740426	A	G	0.053	*CDCA7*	1.21 (1.14–1.28)	1.85E-11	AoU	1.11 (0.92–1.34)	2.78E-01	1.20 (1.14–1.27)	1.46E-11	0.40
MM-MGUS	MM-MGUS	3q23	rs9819371	C	T	0.076	*RASA2*	0.83 (0.78–0.88)	1.30E-09	AoU	0.87 (0.72–1.04)	1.28E-01	0.83 (0.78–0.88)	4.41E-10	0.65
MM-MGUS	MM-MGUS, Cell-P	6q23.3	rs56293029	C	A	0.264	*HBS1L, MYB*	1.11 (1.07–1.15)	4.37E-10	AoU	1.10 (0.99–1.21)	8.25E-02	1.11 (1.07–1.14)	9.57E-11	0.82
MM-MGUS	LN, Cell-P	7p15.3	rs11763022	G	A	0.377	*CDCA7L, DNAH11*	0.87 (0.84–0.89)	1.15E-24	AoU	0.83 (0.75–0.92)	3.16E-04	0.86 (0.84–0.89)	2.20E-27	0.45
MM-MGUS	Cell-P	7q36.1	rs111632177	G	A	0.092	*ABCF2, SMARCD3*	1.18 (1.13–1.23)	7.36E-15	AoU	1.12 (0.97–1.28)	1.23E-01	1.17 (1.13–1.22)	2.95E-15	0.45
MM-MGUS	MM-MGUS	16p11.2	rs3747481	C	T	0.277	*RNF40, TAOK2*	1.10 (1.07–1.14)	7.55E-09	AoU	1.06 (0.96–1.18)	2.43E-01	1.10 (1.06–1.13)	4.59E-09	0.53
MM-MGUS	MM-MGUS, Cell-P	19p13.3	rs11085015	T	G	0.827	*NFIC*	0.85 (0.82–0.89)	4.61E-14	AoU	0.88 (0.78–0.99)	3.06E-02	0.85 (0.82–0.89)	4.76E-15	0.65
MM-MGUS	MM-MGUS, Cell-P	22q13.1	rs139420	C	T	0.251	*CBX7*	1.12 (1.08–1.16)	1.49E-11	AoU	1.13 (1.01–1.25)	2.53E-02	1.12 (1.09–1.16)	1.17E-12	0.89
CLL-MGUS	LN, Soma-G2, Drug-G1	2q37.1	rs2396742	C	T	0.223	*SP140*	1.23 (1.18–1.27)	2.18E-29	AoU	1.16 (1.05–1.28)	4.66E-03	1.22 (1.18–1.26)	6.73E-31	0.30
CLL-MGUS	ASSET	9p13.2	rs12554596	A	G	0.209	*PAX5*	1.12 (1.08–1.16)	7.82E-10	AoU	1.05 (0.94–1.17)	4.08E-01	1.11 (1.07–1.15)	1.08E-09	0.25
CLL-MGUS	ASSET	19q13.12	rs2293688	C	G	0.352	*PSENEN*	0.90 (0.87–0.93)	1.05E-10	AoU	0.91 (0.83–1.00)	4.48E-02	0.90 (0.88–0.93)	1.35E-11	0.87
CLL-MM	Drug-G1, Soma-G2, LN	16q23.1	rs4411525	A	C	0.399	*RFWD3*	0.90 (0.87–0.93)	2.06E-10	AoU	0.99 (0.88–1.12)	8.98E-01	0.91 (0.88–0.93)	6.18E-10	0.14
LN	LN	1q43	rs72755295	A	G	0.038	*EXO1*	1.15 (1.10–1.21)	2.28E-08	PLCO + AoU	1.23 (1.06–1.43)	6.39E-03	1.16 (1.11–1.21)	6.81E-10	0.40
LN	LN, Cell-B	2q24.2	rs35667974	T	C	0.011	*IFIH1*	1.21 (1.14–1.29)	1.83E-09	PLCO + AoU	1.18 (0.98–1.43)	8.00E-02	1.21 (1.14–1.28)	4.02E-10	0.85
LN	LN, Cell-B	3q13.33	rs28876421	G	T	0.352	*CD86*	0.94 (0.93–0.96)	4.38E-09	PLCO + AoU	0.94 (0.89–0.99)	3.04E-02	0.94 (0.93–0.96)	3.76E-10	0.92
LN	LN	3q27.3	rs2253944	A	G	0.325	*LPP*	1.07 (1.05–1.09)	2.55E-12	PLCO + AoU	1.05 (0.99–1.11)	1.24E-01	1.07 (1.05–1.09)	1.01E-12	0.46
LN	LN	4q24	rs5860793	G	GC	0.684	*TET2*	1.06 (1.04–1.08)	3.00E-08	PLCO + AoU	1.02 (0.97–1.08)	4.18E-01	1.05 (1.03–1.07)	3.76E-08	0.30
LN	LN, Cell-B	10q22.1	rs78325861	C	G	0.046	*PRF1*	1.20 (1.14–1.25)	2.35E-15	PLCO + AoU	1.42 (1.24–1.61)	2.07E-07	1.22 (1.17–1.27)	4.78E-20	0.02
LN	LN	11q24.3	rs73013527	C	T	0.530	*ETS1*	0.95 (0.93–0.97)	2.17E-08	PLCO + AoU	0.95 (0.90–1.00)	4.11E-02	0.95 (0.93–0.97)	2.56E-09	0.91
LN	LN	22q12.1	rs9625454	C	T	0.025	*CHEK2, PITPNB, TTC28*	1.16 (1.11–1.22)	2.01E-09	PLCO + AoU	1.07 (0.90–1.27)	4.15E-01	1.16 (1.10–1.21)	2.20E-09	0.38

The table summarizes novel genetic loci discovered via multi-trait association analyses and supported by replication in the PLCO and/or AoU cohorts, with identified contributor subtype(s). The Phenotype column indicates the primary single subtype or multi-trait combination contributing to each association. For each variant, genomic position, alleles, allele frequency (AF), and mapped gene(s) are listed. Odds ratios (OR) with 95% confidence intervals (CI) and corresponding *P*-values are provided for both discovery and replication cohorts, as well as for the combined meta-analysis. The *P*_het_ column reports the heterogeneity *P*-value across studies.

^a^The mapped genes were identified with a combination of various methods (see “Methods”, “Locus to gene mapping” section).

^*^P_het_ < 0.05 indicates significant heterogeneity between studies.

To further explore genetic architecture, we assessed genome-wide genetic correlation (rg) among LN subtypes using cross-trait linkage disequilibrium score regression (LDSC) (Fig. 3c; Supplementary Table 14), revealing a strong genetic correlation between MM and MGUS (rg = 0.75, SE = 0.14, *P* = 5.83 × 10^−^^8^), consistent with their known precursor–disease relationship. A moderate but significant correlation was also observed between MM and CLL (rg = 0.36, SE = 0.13, *P* = 4.6 × 10^−^³), but not between CLL and MGUS (rg = 0.23, SE = 0.19, *P* = 0.079). Additionally, CLL showed significant genetic correlation with the MM-MGUS phenocluster (rg = 0.37, SE = 0.11, *P* = 1.10 × 10^−^³).

### Identified causal variants and genes

We performed fine-mapping using the SuSiE framework to prioritize candidate causal variants across significant loci identified from single-subtype, phenocluster, and multi-trait analyses. In total, 169 fine-mapping regions across various phenotypes yielded 95% credible sets (CS), with coverage consistently ≥ 95% (Supplementary Table 15). The average CS size was nearly 21 variants, though high-confidence signals were observed at several loci with singleton or small sets. (Supplementary Tables 15 and 16).

**Fig. 4:**
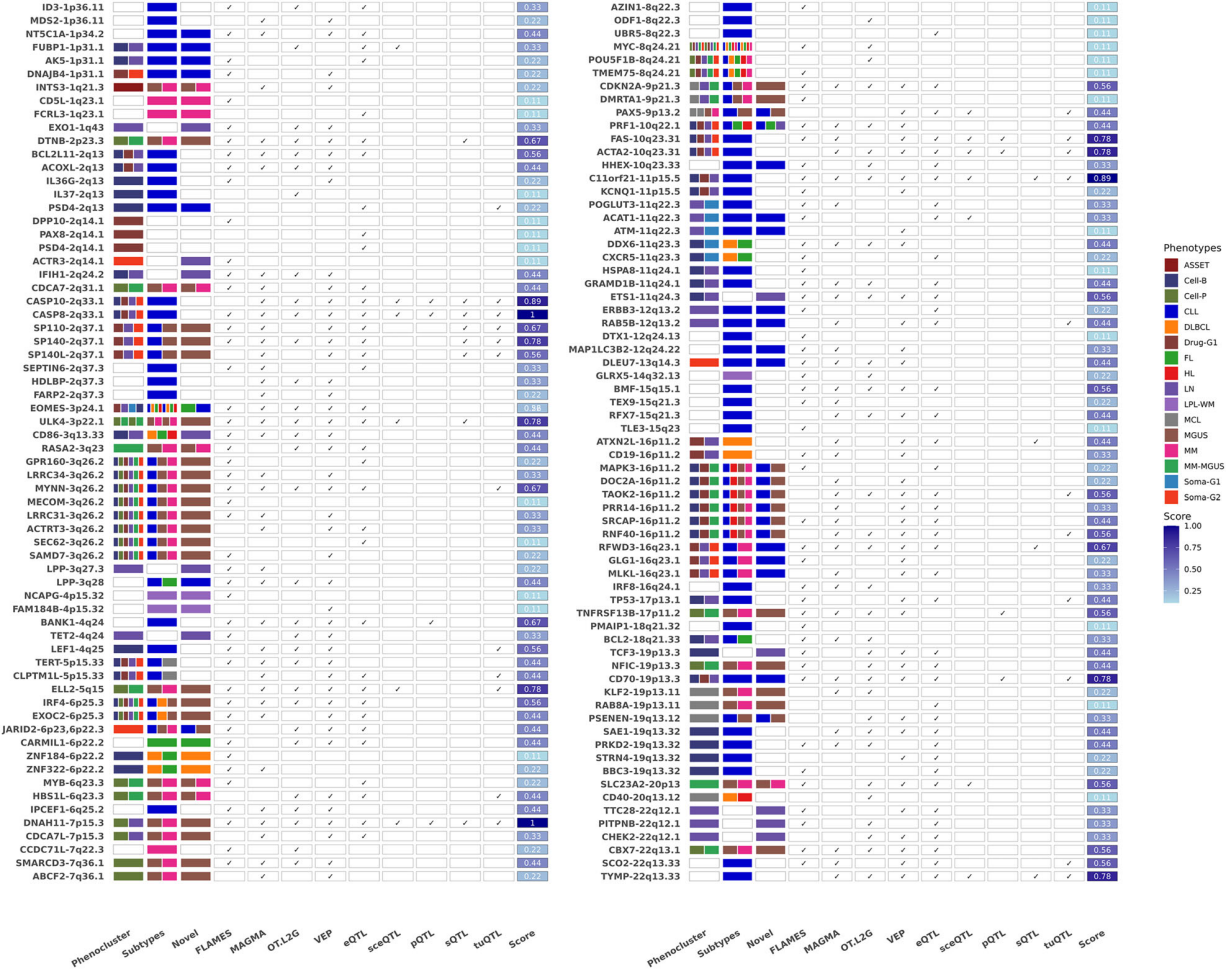
The results of locus to gene mapping. Tile-plot showing the genes identified with different methods. The columns from FLAMES to tuQTL show data sources that support the link to each gene, and the score column shows the average number of supports.

To translate fine-mapped variants into putative effector genes, we integrated variant-to-gene mapping results from the MAGMA-based gene-level association testing (Supplementary Tables 17 and 18), regulatory feature annotation and gene prioritization from FLAMES (Supplementary Table 19), Open Targets’ locus-to-gene scores (Supplementary Table 20), variant functional predictions from Ensembl VEP (Supplementary Table 21), and colocalization with cis-molecular QTLs in blood and hematopoietic cell types (Supplementary Table 22). By integrating evidence from these approaches, we mapped 131 candidate genes across the 169 loci (Supplementary Table 23). Gene assignments were made based on scoring convergence across methods, with summary metrics presented in Supplementary Table 24 and visualized in Fig. 4.

Among the mapped genes, *CASP8, CD70, ELL2, ULK4, SP140*, and *C11orf21* were supported by five or more independent lines of evidence (e.g., FLAMES, SuSiE, MAGMA, molQTLs, and functional annotations), highlighting them as high-confidence candidate genes. Of these, *CASP8* (chr2q33.1) was the only gene to be supported by all methods, including expression and splicing QTLs across multiple tissues, suggesting strong regulatory relevance. Several loci also revealed strong co-localization between fine-mapped SNPs and expression effects, such as *MYNN*, *CDKN2A*, and *CDCA7L*, particularly in blood or hematopoietic-relevant cell types.

### Functional enrichment and druggability analyses

**Fig. 5:**
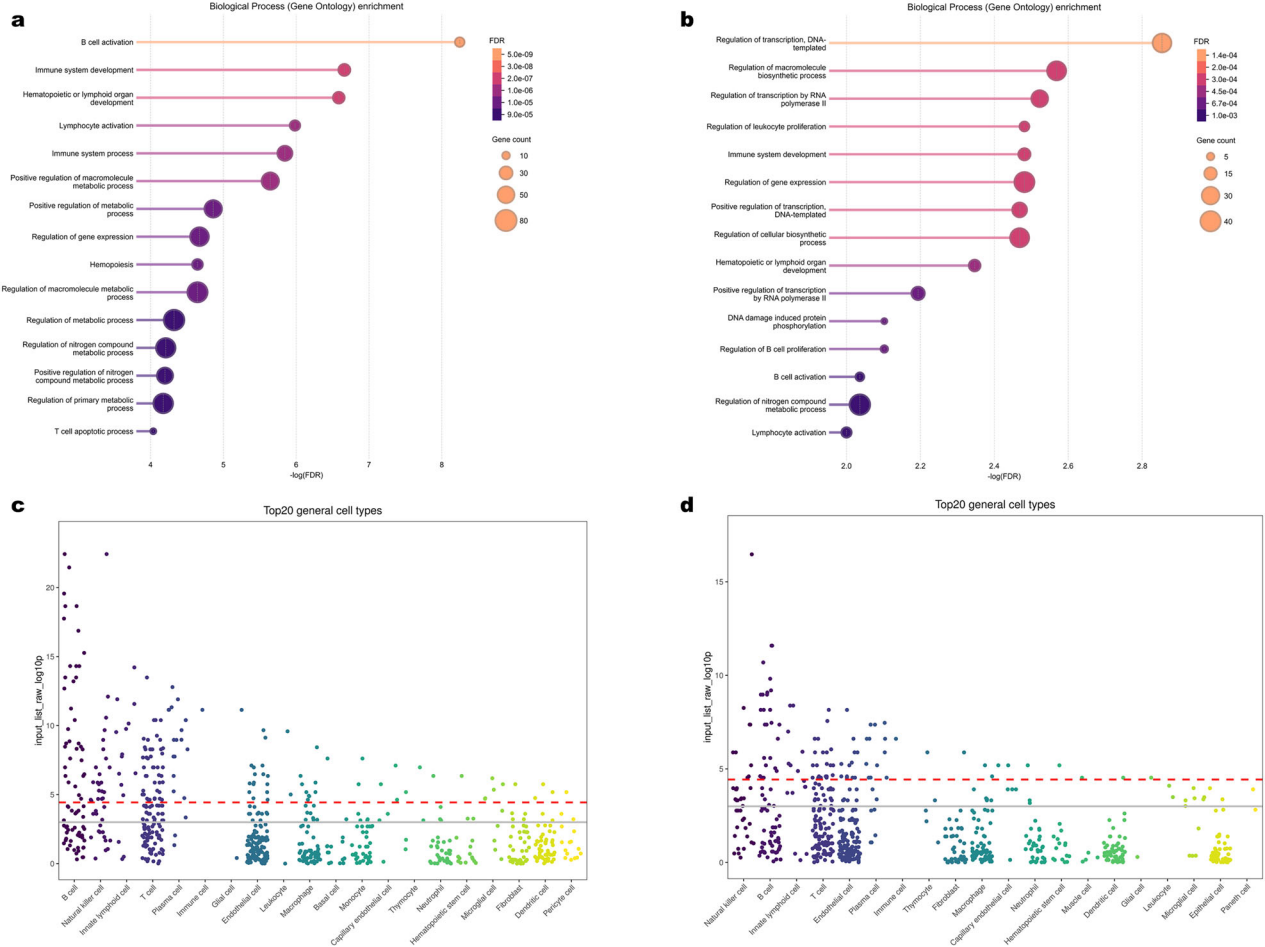
Enrichment of identified genes. **a** The top fifteen significantly enriched GO biological process categories for all identified genes. **b** The top fifteen significantly enriched GO biological process categories for novel identified genes. The horizontal axis shows −log_10_(FDR), and the balloon sizes are proportional to the number of genes. **c** Cell type enrichment results for the top 20 cell types for all identified genes. **d** Cell type enrichment results for the top 20 cell types for novel identified genes. Vertical axis: −log_10_*P*.

To interpret the biological functions of the mapped candidate genes, we conducted Gene Ontology (GO) enrichment analysis for biological processes across both the full set of 131 genes and the subset of 78 genes uniquely mapped to novel loci (Supplementary Table 25). The full gene set revealed strong enrichment in immune-related pathways, transcriptional regulation, and apoptosis (Supplementary Table 25 and Fig. 5a). Additionally, there was significant enrichment for terms related to transcriptional control and programmed cell death. Focusing specifically on the novel loci, the subset of 78 genes exhibited a similar but distinct pattern, (Supplementary Table 25 and Fig. 5b), suggesting additional mechanistic contributions to genome stability, transcriptional control, cell cycle progression, and B cell-specific functions.

To evaluate the cellular context of the identified genes, we applied single-cell enrichment analysis using WebCSEA on both the complete set of 131 prioritized genes (Supplementary Table 26 and Fig. 5c) and the subset of 78 genes mapped exclusively to novel loci (Supplementary Table 26 and Fig. 5d). We observed a strong and consistent enrichment in immune cell populations, especially B cells and plasma cells, across a wide range of anatomical contexts. Additional enrichment was observed for T cells, innate lymphoid cells, natural killer (NK) cells, and leukocytes.

To assess the therapeutic relevance of the mapped genes, we performed druggability analysis using DGIdb and ATC code enrichment. DGIdb analysis revealed 1,358 gene-drug interaction pairs involving known or investigational compounds (Supplementary Table 27), with 453 of these interactions linked to ATC-classified drugs. ATC enrichment analysis (Supplementary Table 28) demonstrated significant overrepresentation of antineoplastic and immunomodulating agents (Level 1: L, OR = 9.49, *P* = 6.8 × 10^−^^6^; Level 2: L01, OR = 13.8, *P* = 7.7 × 10^−^^7^), strongly supporting the clinical actionability of prioritized genes in oncology and immunotherapy (Supplementary Table 29). These findings suggest that many mapped genes, especially those from novel loci, are not only biologically relevant but also represent plausible candidates for therapeutic targeting or drug repurposing. In addition to conventional therapeutic agents, our DGI analysis identified several environmental and industrial compounds with potential relevance to lymphoid malignancies. Notably, we observed a high-scoring interaction between the product of *PAX5*, a novel CLL-MGUS locus, and 2,3,7,8-tetrachlorodibenzo-*p*-dioxin (TCDD), a known environmental pollutant and the principal toxic component of Agent Orange. TCDD has been previously implicated in lymphomagenesis through its immunotoxic effects and disruption of B-cell development [42], and reported exposure to TCDD has been linked to MGUS to MM progression [43]. Moreover, the identified risk variant, rs12554596, is a reported eQTL of *PAX5* in lymphoblastoid cell lines in multiple sources (Supplementary Table 22), supporting its regulatory impact.

By using the Open Target Platform, identified approved or trial-stage compounds for 16 prioritized genes (Supplementary Table 30), further expanding the translational potential of our findings. Sixteen gene products were identified as targets of drugs either approved or in clinical trials (Table 3), highlighting their potential clinical relevance in LN and other malignancies. Among these, CD19 is the most extensively targeted, with multiple approved therapies used in the treatment of B-cell malignancies. TP53 and BCL2 also have several small-molecule inhibitors and oligonucleotide drugs under investigation or approved in hematologic cancers. ERBB3 is targeted by a variety of antibody-based and small-molecule inhibitors, mainly in solid tumors, but with potential implications for LN. Other genes such as MAPK3, CD70, CD40, CHEK2, and PSENEN are being explored in clinical trials, often in broader oncologic contexts. Notably, several drug-gene pairs—including those targeting CASP8, CASP10, KCNQ1, and HSPA8—are not currently approved nor under clinical evaluation for LN, representing investigational candidates with potential for future development in hematologic malignancies.

**Table 3:** Drugs targeting putative LN risk genes.

Cytoband	Gene(s)	Drugs	Mechanism of action	Drug type	Approved	Clinical trial
2q33.1	*CASP10, CASP8*	Emricasan, nivocasan	Caspase inhibitor	Small molecule	No	No
3q13.33	*CD86*	Abatacept, belatacept	CD86 inhibitor	Protein	No	MM
5p15.33	*TERT*	Imetelstat	TERT inhibitor	Oligonucleotide	MDS	MM, lymphomas, MDS
11p15.5	*KCNQ1*	Dalfampridine, guanidine, amifampridine, nerispirdine, tedisamil	Voltage-gated potassium channel blocker	Small molecule	No	No
11p15.5	*KCNQ1*	Ezogabine	KCNQ (Kv7) potassium channel opener	Small molecule	No	No
11q24.1	*HSPA8*	Forigerimod	HSPA8 inhibitor	Protein	No	No
**12q13.2**	***ERBB3***	**Istiratumab, cdx-3379, av-203, seribantumab, patritumab, duligotuzumab, lumretuzumab**	**erbB-3 inhibitor**	**Antibody**	**No**	**Solid tumors**
**12q13.2**	***ERBB3***	**Tarloxotinib, vandetanib, poziotinib**	**Epidermal growth factor receptor inhibitor**	**Small molecule**	**Thyroid cancer (vandetanib)**	**MM, solid tumors**
**12q13.2**	***ERBB3***	**Patritumab deruxtecan**	**erbB-3 binding agent**	**Antibody drug conjugate**	**No**	**Solid tumors**
**12q13.2**	***ERBB3***	**MM-111**	**ErbB-2/ErbB-3 heterodimer inhibitor**	**Unknown**	**No**	
16p11.2	*CD19*	Coltuximab ravtansine, loncastuximab tesirine, denintuzumab mafodotin	CD19 binding agent	Antibody drug conjugate	DLBCL (loncastuximab)	NHL, B-cell lymphoma
16p11.2	*CD19*	Obexelimab	CD19 inhibitor	Antibody	No	No
16p11.2	*CD19*	MDX-1342, tafasitamab, inebilizumab	CD19 binding agent	Antibody	DLBCL (tafasitamab)	CLL, MM, DLBCL, MCL, FL, ALL
16p11.2	*CD19*	Lisocabtagene maraleucel, axicabtagene ciloleucel, brexucabtagene autoleucel	CD19 binding agent	Gene	CLL, DLBCL, FL (lisocabtagene), DLBCL (axicabtagene), MCL, ALL (brexucabtagene)	Lymphomas and leukemias
16p11.2	*CD19*	Blinatumomab	CD19 cross-linking agent	Antibody	ALL	DLBCL, MM, BL
16p11.2	*CD19*	Tisagenlecleucel	CD19 binding agent	Cell	FL, ALL, DLBCL	CLL, MM, pancreatic carcinoma
**16p11.2**	***MAPK3***	**Ravoxertinib, ulixertinib**	**MAP kinase ERK1 inhibitor**	**Small molecule**	**No**	**AML, MDS, solid tumors**
**16p11.2**	***MAPK3***	**Temuterkib**	**MAP kinase; ERK1/ERK2 inhibitor**	**Small molecule**	**No**	**AML, solid tumors**
17p13.1	*TP53*	Cenersen, teprasiran	p53 mRNA RNAi inhibitor	Oligonucleotide	No	CLL, AML, MDS (cenersen)
17p13.1	*TP53*	Eprenetapopt	Cellular tumor antigen p53 stabilizer	Small molecule	No	MCL, MDS, solid tumors
17p13.1	*TP53*	Alrizomadlin, idasanutlin, navtemadlin	p53/oncoprotein Mdm2 inhibitor	Small molecule	No	DLBCL, MM, CLL, CML, AML, MDS, solid tumors
17p13.1	*TP53*	Contusugene ladenovec	Cellular tumor antigen p53 exogenous gene	Gene	No	Solid tumors
18q21.33	*BCL2*	Obatoclax, lisaftoclax, navitoclax, venetoclax	Apoptosis regulator Bcl-2 inhibitor	Small molecule	CLL, AML (venetoclax)	Lymphomas, leukemias, MM, MDS, AML, solid tumors
18q21.33	*BCL2*	Oblimersen	Bcl-2 mRNA antisense inhibitor	Oligonucleotide	No	CLL, DLBCL, MM, MCL, LPL-WM, FL, solid tumors
**19p13.3**	***CD70***	**MDX-1411**	**CD70 antigen cross-linking agent**	**Antibody**	**No**	**MCL**
**19p13.3**	***CD70***	**Cusatuzumab**	**CD70 antigen inhibitor**	**Antibody**	**No**	**AML, CMML, MDS**
**19p13.3**	***CD70***	**Vorsetuzumab mafodotin**	**CD70 antigen-binding agent**	**Antibody drug conjugate**	**No**	**NHL, renal cell carcinoma**
**19q13.12**	***PSENEN***	**Tarenflurbil, rg-4733, nirogacestat, avagacestat, begacestat, semagacestat**	**Gamma-secretase modulator**	**Small molecule**	**No**	**MM, solid tumors**
**20q13.12**	***CD40***	**Sotigalimab, selicrelumab, cdx-1140, giloralimab**	**CD40 agonist**	**Antibody**	**No**	**MCL, DLBCL, solid tumors**
**20q13.12**	***CD40***	**Iscalimab, bleselumab, dacetuzumab**	**CD40 inhibitor**	**Antibody**	**No**	**CLL, NHL, MM, DLBCL (dacetuzumab)**
**20q13.12**	***CD40***	**Lucatumumab**	**CD40 antagonist**	**Antibody**	**No**	**FL, MM, CLL, HL**
**22q12.1**	***CHEK2***	**Prexasertib, xl-844**	**CHEK2 inhibitor**	**Small molecule**	**No**	**CLL (XL-844), CML, MDS, AML, solid tumors**
22q13.33	*TYMP*	Tipiracil	TYMP inhibitor	Small molecule	Colorectal cancer	Solid tumors

The list of drugs that target identified candidate genes/proteins from the OpenTarget and DrugBank database. The drugs are grouped by mechanism of action and drug type, and approval and clinical trial status for neoplasms are listed. The novel identified risk genes are marked in bold. *ERBB3* and *CD70* are novel for CLL, *PSENEN* is novel for MGUS and CLL, and *CHEK2* is novel for the LN phenocluster.

## Discussion

This study represents one of the most comprehensive germline investigations of LNs to date, integrating large-scale genome-wide association analyses across multiple cohorts, phenotypic clustering, fine-mapping, functional annotation, and therapeutic target discovery. Our integrative approach revealed 76 GWS loci associated with individual LN subtypes and phenoclusters, including 20 replicated novel loci, 19 of which associated with risk of individual subtypes and 12 multi-trait loci. These findings substantially expand the known genetic architecture of LNs and offer critical insights into subtype-specific and pleiotropic susceptibility mechanisms. Importantly, they also uncover biological mechanisms central to lymphomagenesis and highlight multiple avenues for clinical translation.

A key innovation of this study is the use of a hierarchical phenocluster strategy, which allowed us to transcend traditional histopathologic boundaries by identifying clusters of LNs with shared genetic architectures. By incorporating phenotypic clustering with multi-trait colocalization and GWAS resolution, we extended these insights to include precursor conditions such as MGUS and rarer subtypes like LPL-WM, thereby offering broader biological context and novel subtype-specific loci. Some loci showed high PP for colocalization with multiple subtypes, supporting true biological pleiotropy, while others appeared subtype-specific. CLL emerged as a major contributor to many multi-trait associations, which could reflect either true biological pleiotropy or be driven by its relatively higher GWAS heritability and statistical power compared to other subtypes. This phenocluster-informed approach provides a biologically grounded framework to interpret shared susceptibility and reveals etiologic commonalities across clinically distinct LNs.

We prioritized 131 candidate risk genes, supported by regulatory annotations, molQTLs, and gene-level association statistics. These genes were significantly enriched in pathways related to B-cell differentiation, transcriptional regulation, DNA repair, and immune signaling—core processes known to underpin lymphomagenesis. Consistent with previous findings from Went et al., our results reinforce the role of early B-cell developmental genes such as *PAX5* and transcriptional regulators such as *BCL11A* and *IRF4*, the latter having pleiotropic effects across multiple hematologic malignancies [44]. Notably, single-cell enrichment analyses localized the expression of prioritized genes to naive and memory B cells, plasma cells, and innate lymphoid populations, echoing observations in recent transcriptomic studies that implicate these compartments in both disease initiation and progression. Thus, our results offer direct insight into the specific immune cell contexts in which germline variation may exert pathogenic effects.

Genetic correlation analysis further underscored biological connections among LNs. We observed genome-wide correlation between CLL and MM as previously reported [4, 5], despite their distinct clinical profiles. This suggests convergent germline mechanisms that may be therapeutically exploitable. The exceptionally high correlation between MM and MGUS aligns with their known precursor-product relationship. These findings are in line with recent cross-trait studies that identified overlapping heritability patterns between plasma cell and lymphoid disorders, particularly within immune regulatory loci such as *ELL2*, *TNFRSF13B*, and *PRKD2* [5, 44]. Together, these results reinforce the hypothesis that immune-related pathways are key determinants of both shared and divergent risk across LN subtypes.

Beyond risk genes, our analysis explored the translational potential of risk genes through DGI mapping. We identified over 1200 DGIs, including 453 involving agents classified under the ATC system, with significant enrichment for antineoplastic and immunomodulatory therapies. In particular, we found that 16 gene products are targets of drugs either approved or in clinical trials. This aligns with previous work demonstrating the therapeutic relevance of germline GWAS loci in hematologic cancers [45, 46]. We also identified interactions between risk genes and environmental toxicants. Of particular interest is *PAX5*, a novel locus associated with both CLL and MGUS, which showed a high-confidence interaction with TCDD—the toxic component of Agent Orange. TCDD has long been implicated in immune dysregulation and lymphomagenesis and was recently shown to increase the risk of MGUS-to-MM progression in a large cohort of Vietnam-era U.S. veterans [43]. Given PAX5’s central role in B-cell development, one plausible hypothesis is that TCDD exposure may alter B-cell maturation or promote genomic instability in progenitor cells via modulation of AHR (aryl hydrocarbon receptor) signaling, thereby interacting with germline variants to enhance susceptibility to transformation. These findings point to a potential gene–environment axis in LN risk and warrant further mechanistic investigation. They also suggest that inherited variation at immune developmental genes may modify individual responses to environmental exposures—a concept with potential implications for public health and precision prevention.

While our study offers several strengths—including large sample size, robust statistical methodology, and integration of regulatory and pharmacogenomic annotations—it also has limitations. All analyses were restricted to individuals of European ancestry, limiting generalizability to non-European populations and potentially missing population-specific variants. Disease phenotyping, although harmonized across biobanks, is subject to variability in diagnostic coding and clinical ascertainment, which may introduce misclassification bias. Furthermore, the lack of individual-level data in MVP and FinnGen constrained our ability to conduct uniform fine-mapping and joint modeling across all cohorts. Finally, while our environmental and chemical interaction findings are compelling, they remain hypothesis-generating and require further mechanistic validation.

In conclusion, this study reports 20 novel subtype-specific and shared genetic risk factors for LNs. By linking genetic risk loci to candidate genes, we highlight their biological relevance—connecting them to immune cell biology, drug targets, and environmental exposures. Our results not only inform disease etiology but also highlight pathways and genes with clear clinical relevance, offering a resource for future functional studies and a roadmap for potential therapeutic development in lymphoid malignancies.

## Supplementary information

Supplementary Information

Supplementary Tables

Supplementary Data1 and 2

## Data Availability

UK Biobank data (genotypes and phenotypes) are available under controlled access (application number 66591). GWAS summary statistics from this study will be made available through the GWAS Catalog (accession codes: GCST90624736-GCST90624750). As the GWAS Catalog does not support analysis-specific outputs from meta-analyses—such as effect direction and heterogeneity statistics—we have deposited the complete raw outputs from the meta-analysis, as well as the ASSET results, in Zenodo. These data are publicly accessible at DOI: 10.5281/zenodo.15464477. MVP GWAS summary statistics are available in dbGaP (phs002453). FinnGen v12 summary statistics are available at: https://www.finngen.fi/en/access_results. All of Us GWAS summary statistics are accessible to registered users at: https://workbench.researchallofus.org. PLCO summary statistics are publicly accessible at: https://exploregwas.cancer.gov/. Somatic mutation data are available via cBioPortal: https://www.cbioportal.org/, and the derived mutation–LN subtype matrix is provided in Supplementary Data 1. Approved drug data were obtained from Open Targets: https://www.opentargets.org/; the derived drug–LN subtype matrix is in Supplementary Data 2. UK Biobank LD data were accessed at: https://registry.opendata.aws/ukbb-ld/. The complete analysis workflow, scripts, and visualization tools are available in the GitHub repository: https://github.com/biomguler/LN_Phenocluster.
