# Supplementary material for: Clustering of lymphoid neoplasms by cell of origin, somatic mutation and drug usage profiles: a multi-trait genome-wide association study
Source: Blood Cancer J. 2025 Aug 29;15(1):147. doi: 10.1038/s41408-025-01351-4 (PMC12397487; doi:10.1038/s41408-025-01351-4)
Supplement: Supplementary file 1 — Supplementary Information [file 41408_2025_1351_MOESM1_ESM.docx]

**Supplementary Information**

**to**

**Title: Clustering of Lymphoid Neoplasms by Cell of Origin, Somatic Mutation and Drug Usage Profiles: A Multi-trait Genome-Wide Association Study**

**Running Title: Pleiotropic risk loci in lymphoid neoplasms**

Murat Güler^1, 2^, Federico Canzian^1^

^1^ Genomic Epidemiology Group, German Cancer Research Center (DKFZ), Heidelberg, Germany

^2^ Medizinische Fakultät Heidelberg, Universität Heidelberg

**ORCID iD (email address)**

MG 0000-0003-4700-3842 ([murat.guler@dkfz-heidelberg.de](mailto:murat.guler@dkfz-heidelberg.de))

FC 0000-0002-4261-4583 ([f.canzian@dkfz-heidelberg.de](mailto:f.canzian@dkfz-heidelberg.de))

[**Description and content** 3](#_Toc203640918)

[**Summary List of Supplementary Tables** 4](#_Toc203640919)

[**Summary List of Supplementary Figures** 4](#_Toc203640920)

[**List of Supplementary Data** 4](#_Toc203640921)

[**1. Hierarchical Clustering of LNs** 5](#_Toc203640922)

[**3. UK Biobank study population, genetic data, GWAS and post-GWAS** 9](#_Toc203640923)

[**4. FinnGen** 11](#_Toc203640924)

[**5. Million Veteran Program (MVP)** 11](#_Toc203640925)

[**6. Replication in All of Us (AoU) and PLCO cohorts** 13](#_Toc203640926)

[**7. Meta-analysis of individual subtypes** 14](#_Toc203640927)

[**8. Meta-analysis of phenoclusters** 16](#_Toc203640928)

[**9. ASSET** 19](#_Toc203640929)

[**10. Identification of driver and contributor subtypes from multi-trait** 21](#_Toc203640930)

[**11. LDSC** 22](#_Toc203640931)

[**12. MAGMA set-based analysis** 22](#_Toc203640932)

[**13. Locus to gene mapping** 23](#_Toc203640933)

[**14. Risk Gene and Enrichment Analysis** 24](#_Toc203640934)

[**Supplementary References** 26](#_Toc203640935)

# **Description and content**

This document provides detailed descriptions of the supplementary methods, figures, and analyses referenced in the main manuscript. Supplementary tables and data files are available as separate Excel workbooks—“Supplementary_tables.xlsx” and “Supplementary_data.xlsx.” A comprehensive list of these materials, along with brief descriptions, is included below.

To facilitate tracking of the identified loci, we assigned a unique index to each locus. This index is consistently used across all supplementary tables that contain information about a given locus. Each locus index encodes the genomic region (defined as a 500 kb window) and follows an alphanumeric format starting with “R.” Loci were defined using PLINK clumping with the following parameters: --clump-p1 5e-8 --clump-p2 5e-8 --clump-r2 0.01 --clump-kb 10000.

All code and computational pipelines used to perform the analyses and generate figures are publicly accessible in the following GitHub repository: <https://github.com/biomguler/LN_Phenocluster/>

## **Summary List of Supplementary Tables**

| **Section** | **Supplementary Tables** | **Description** |
| --- | --- | --- |
| **A. Cohorts and Case Counts** | Table S01–S03 | Case selection criteria, population summaries (UKB, FinnGen, MVP) |
|  | Table S04 | Case/control counts across cohorts and subtypes |
|  | Table S07 | Genomic inflation factors (λgc) |
| **B. Single-Subtype GWAS Results** | Table S05 | Previously reported loci |
|  | Table S06 | Significant and suggestive associations (single-subtype GWAS) |
| **C. Multi-Trait and Phenocluster GWAS** | Table S08 | Phenocluster-based GWAS results |
|  | Table S09 | ASSET analysis (significant/suggestive) |
|  | Table S10 | HyPrColoc colocalization results |
|  | Table S11–S12 | Subtype contributions and pleiotropy summary |
|  | Table S13 | Replication of multi-trait loci |
|  | Table S14 | LDSC genetic correlation results |
| **D. Fine-Mapping and Gene Mapping** | Table S15–S16 | SuSiE fine-mapping and credible sets |
|  | Table S17–S18 | MAGMA gene-level association results |
|  | Table S19 | FLAMES gene prioritization |
|  | Table S20 | Open Targets locus-to-gene scores |
|  | Table S21 | Ensembl VEP variant annotations |
|  | Table S22 | Molecular QTLs (blood and immune tissues) |
|  | Table S23–S24 | Final gene lists and gene–locus summary |
| **E. Functional and Translational Annotation** | Table S25 | GO enrichment (all and novel-locus genes) |
|  | Table S26 | Cell-type enrichment (WebCSEA) |
|  | Table S27 | Drug–gene interaction results (DGIdb) |
|  | Table S28–S29 | ATC-coded drugs and enrichment analysis |
|  | Table S30 | Drugs with clinical relevance (Open Targets) |

## **Summary List of Supplementary Figures**

| **Section** | **Supplementary Figures** | **Description** |
| --- | --- | --- |
| **A. Clustering and Phenotype Visualization** | Figure S1–S2 | Hierarchical clustering method comparison |
|  | Figure S3–S4 | Clustering heatmaps (somatic mutations, drugs) |
|  | Figure S5 | MVP phenotype overlap diagram |
| **B. GWAS Manhattan & QQ Plots – Subtypes** | Figure S6–S13 | Subtype-specific Manhattan and QQ plots |
| **C. GWAS Manhattan Plots – Phenoclusters** | Figure S14–S20 | Phenocluster and composite trait GWAS plots |
|  | Figure S21–S22 | ASSET and multi-trait Manhattan plots |
| **D. Enrichment Visualization** | Figure S23 | ATC enrichment (levels 1 and 2) |

## **List of Supplementary Data**

**Supplementary Data 1:** Binary matrix of somatically mutated genes in LNs

**Supplementary Data 2:** Binary matrix of approved drugs for LNs

# **1. Hierarchical Clustering of LNs**

Data for somatic mutation patterns and approved drug usage were obtained from cBioPortal and the Open Targets Platform, respectively (Supplementary Data 1 and 2). For drug data, each LN subtype was queried on the Open Targets platform, and candidate drugs (limited to phase 3 or 4 trials) were manually verified using public databases to confirm clinical approval by the FDA or EMA. A binary matrix (0,1) was constructed to indicate whether a drug was approved for each LN subtype.

For somatic mutations, we downloaded subtype-level data from cBioPortal, capturing genes reported as mutated without filtering by mutation frequency or position. This information was used to construct a binary matrix (0,1), representing the presence or absence of somatic mutations per gene for each LN subtype. Due to computational constraints, analysis was limited to the 10,000 genes mutated in more than 20% of subtypes.

Hierarchical clustering was performed using the hclust function from the R stats package (1), and visualized with the dendextend (2) and gplots (3) packages. To evaluate the most appropriate clustering strategy, we compared eight standard hierarchical clustering methods: “ward.D”, “ward.D2”, “single”, “complete”, “average” (UPGMA), “mcquitty” (WPGMA), “median” (WPGMC), and “centroid” (UPGMC). We assessed their performance using cophenetic correlation coefficients and the Fowlkes–Mallows Index across different cluster counts (k = 3, 4, 5) (4).

These comparisons were conducted using a custom R script (00_Compare_hclust_method.R), which generated correlation plots and calculated performance metrics for each method. Supplementary Figures 1 and 2 illustrate similarities and differences among clustering approaches for somatic mutation and drug-based data, respectively.

Based on these comparisons, we selected Ward’s method combined with the Jaccard similarity coefficient (via dist(method = "binary") in R) for downstream analysis. We then used the 01_LNcluster.R script to generate final phenoclusters and visualize results. The resulting dendrograms were displayed as heatmaps using heatmap.2 from the gplots package, showing the relationships between LN subtypes and their corresponding somatic mutation or drug profiles.

Final clustering outputs are shown in Supplementary Figure 3 (somatically mutated gene-based clusters) and Supplementary Figure 4 (approved drug-based clusters).

**
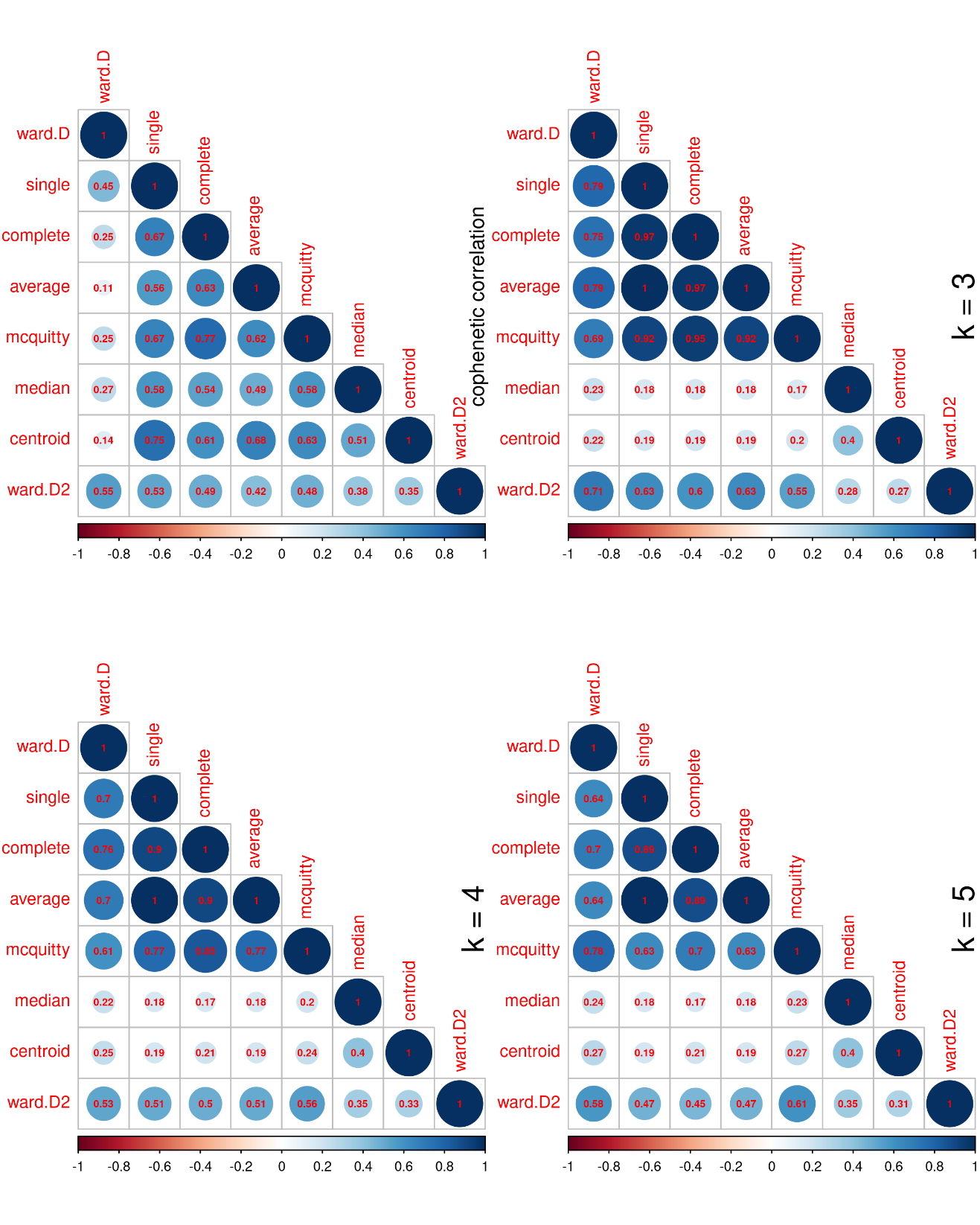
**

**Supplementary Figure 1.** Correlation plots illustrating the similarity/difference between somatically mutated gene-based hierarchical clustering methods. The first plot shows cophenetic correlation (Pearson’s correlation coefficient) between different methods. The other three plots show Fowlkes-Mallows Index based on different number of clusters (k=3, k=4, k=5).


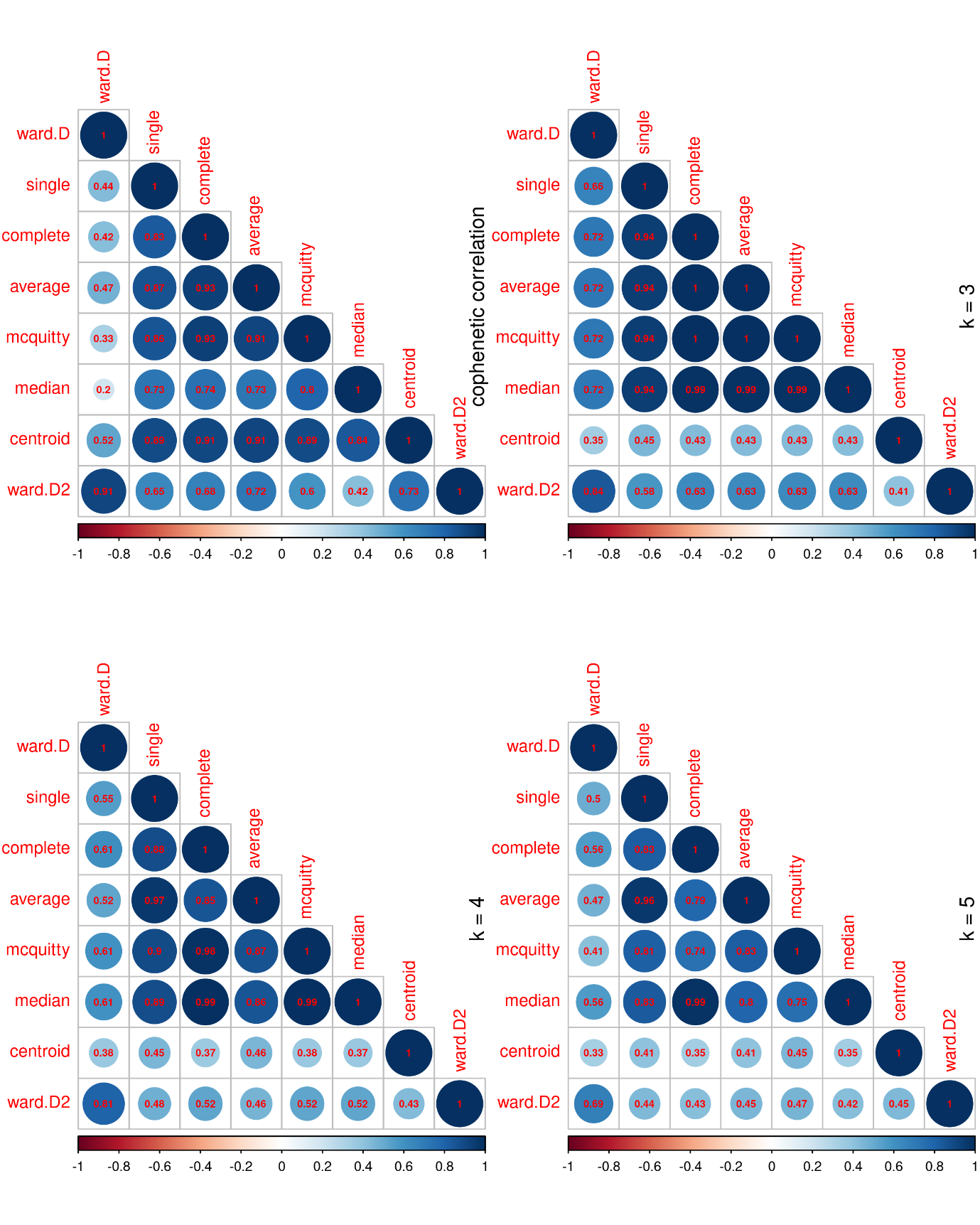


**Supplementary Figure 2.** Correlation plots illustrating the similarity/difference between approved drug-based hierarchical clustering methods. The first plot shows cophenetic correlation (Pearson’s correlation coefficient) between different methods. The other three plots show Fowlkes-Mallows Index based on different number of clusters (k=3, k=4, k=5).


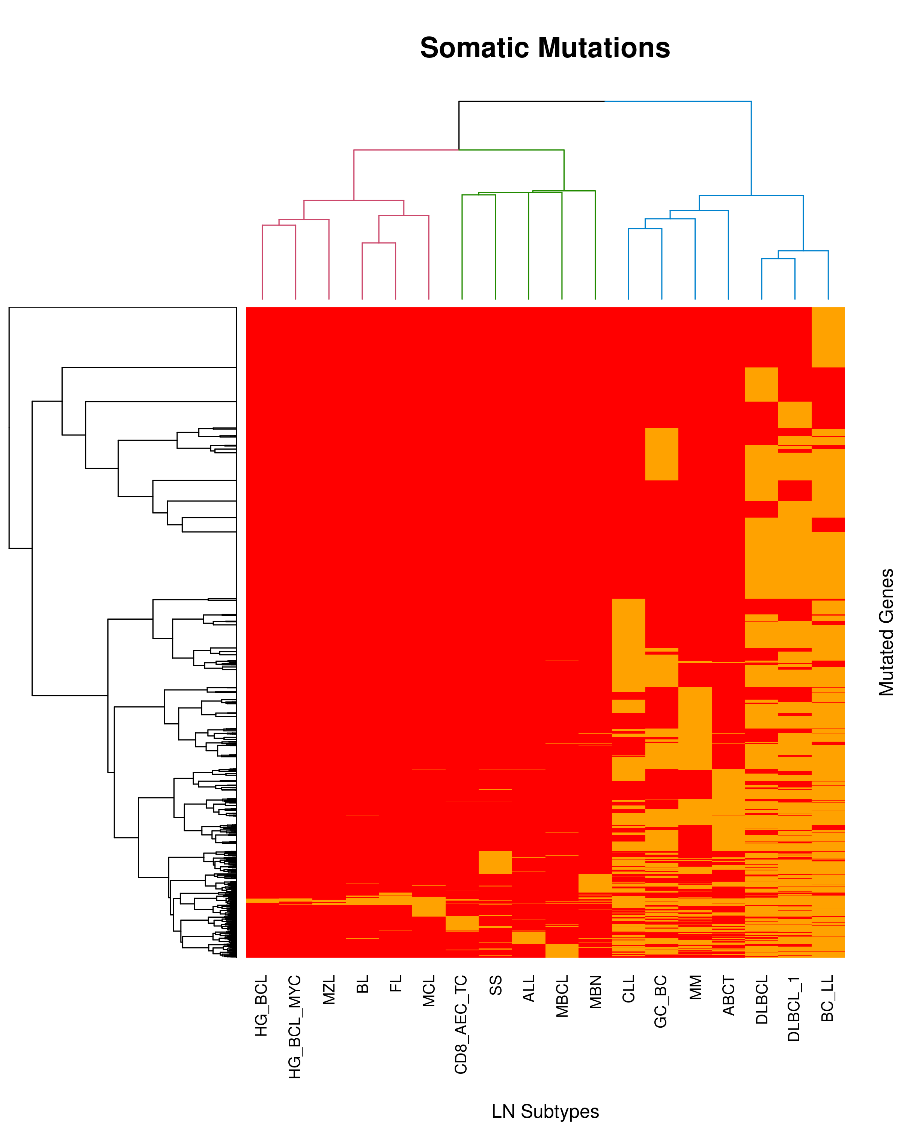


**Supplementary Figure 3.** Heatmap illustrating the somatically mutated gene-based hierarchical clusters of lymphoid neoplasms (LN). On the dendrogram on the left side each branch indicates a gene, and on the top LN subtypes. The red color shows absence of somatic mutation and orange presence of mutation in the gene. The color of upper dendrogram shows groups, the pink, blue and green colors represent Soma-G1, Soma-G2 and Soma-G3, respectively. The abbreviations of subtypes accessed via cBioportal are ABCT: Activated B-cell Type, ALL: Acute Lymphoid Leukemia, AML: Acute Myeloid Leukemia, BC_ALL: B-Cell Acute Lymphoid Leukemia, BC_LL: B-Lymphoblastic Leukemia/Lymphoma, BL: Burkitt Lymphoma, CLL: Chronic Lymphocytic Leukemia/Small Lymphocytic Lymphoma, DLBCL: Diffuse Large B-Cell Lymphoma, DLBCL_1: Diffuse Large B-Cell Lymphoma, NOS, FL: Follicular Lymphoma, GC_BC: Germinal Center B-Cell Type, HG_BCL: High-Grade B-Cell Lymphoma, NOS, HG_BCL_MYC: High-Grade B-Cell Lymphoma, with MYC and BCL2 and/or BCL6 Rearrangements, MCL: Mantle Cell Lymphoma, MZL: Marginal Zone Lymphoma, MBN: Mature B-Cell Neoplasms, MBCL: Monoclonal B-Cell Lymphocytosis, MM: Plasma Cell Myeloma, CD8_AEC_TC: Primary Cutaneous CD8 Positive Aggressive Epidermotropic Cytotoxic T-Cell Lymphoma, SS: Sezary Syndrome.

**
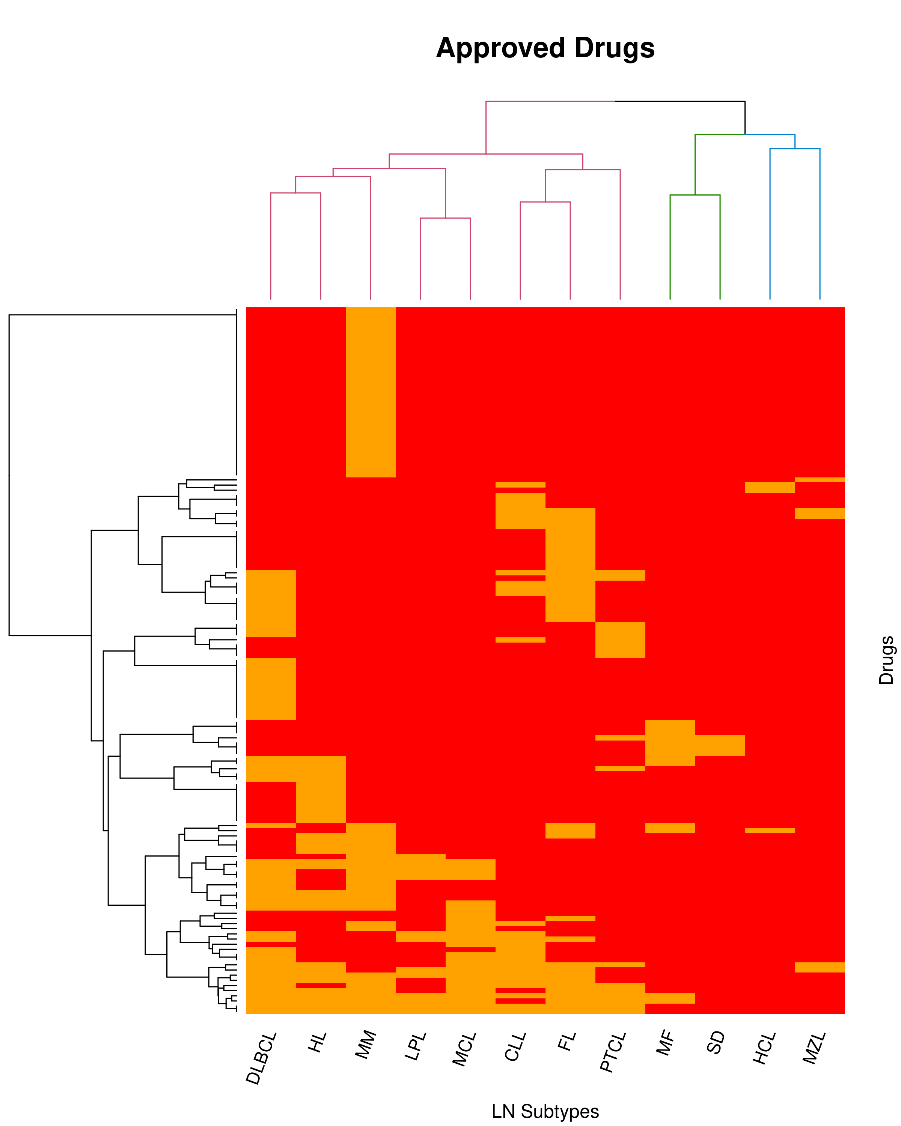
**

**Supplementary Figure 4.** Heatmap illustrating the approved drug-based hierarchical clusters of lymphoid neoplasms (LN). On the dendrogram on the left side each branch indicates a drug and on the top LN subtypes. The red color shows absence of approved drug and orange presence of approved drug. The color of upper dendrogram shows groups, the pink, green and blue colors represent Drug-G1, Drug-G2 and Drug-G3, respectively. The abbreviation subtypes are CLL: Chronic Lymphocytic Leukemia/Small Lymphocytic Lymphoma, DLBCL: Diffuse Large B-Cell Lymphoma, FL: Follicular Lymphoma, HCL: Hairy Cell Leukemia, HL: Hodgkin Lymphoma, LPL: Lymphoplasmacytic Lymphoma, MCL: Mantle Cell Lymphoma, MF: Mycosis Fungoides, MGUS: Monoclonal Gammopathy of Undetermined Significance, MM: Multiple Myeloma, MZL: Marginal Zone Lymphoma, PTCL: Peripheral T-Cell Lymphoma, SD: Sézary Disease.

# **3. UK Biobank study population, genetic data, GWAS and post-GWAS**

The study was conducted using data from the UK Biobank (UKB) resource under Application Number 66591. Both cases and controls were selected from the UKB, a large, population-based prospective cohort study comprising 502,389 participants aged 37 to 69 years at the time of recruitment. Participants were identified through NHS central registers and invited to join the study between 2006 and 2010, with eligibility limited to those residing within 25 miles of one of the 22 assessment centers across England, Wales, and Scotland. Further details of the study protocol can be found elsewhere (5).

Blood samples from UK Biobank participants were collected, and DNA was extracted over an 18-month period between November 2013 and April 2015. A total of 488,377 participants were genotyped using highly similar genotyping arrays in 106 sequential batches. The majority, 438,427 participants, were genotyped with the Applied Biosystems UK Biobank Axiom Array, while 49,950 participants were genotyped with the UK Biobank Lung Exome Variant Evaluation (UK BiLEVE) Array. The UK BiLEVE and UK Biobank Axiom arrays contain 807,411 and 825,927 markers, respectively, with a 95% overlap in marker content (6).

We applied strict inclusion criteria to create a cohort from the entire UK Biobank dataset to minimize missing data and avoid biased results. Participants were excluded if data were unavailable for any of the following: genetic sex (Data-Field 22001), age at cancer diagnosis for lymphoid neoplasms (LN) cases (Data-Field 40008), age at the time of attending the assessment center for controls (Data-Field 21003), ethnic background (Data-Field 21000), genetic ethnic grouping (Data-Field 22006), genetic principal components (Data-Field 22009), or genotype calls (Data-Field 22418). After applying these filters for missing data, 463,863 individuals remained available for selecting LN cases and non-cancer controls.

LN cases were identified using Cancer Registry data (Category 100092) in conjunction with the type of cancer (ICD-10 code, Data-Field 40006), tumor histology (Data-Field 40011), and tumor behavior (Data-Field 40012). LN cases were ascertained based on the InterLymph hierarchical classification of lymphoid neoplasms. The ICD-10 and ICD-O-3 codes for each LN subtype are provided in Supplementary Table 1.

Control selection criteria included the absence of any cancer diagnosis code (Chapter II: Neoplasms) in Cancer Registry data (Category 100092), hospital records (Data-Field 41270), self-reported cancer (Data-Field 20001), or primary cause of death (Data-Field 40001).

We applied quality-control both at the level of individuals and genetic markers. Participants were removed if they have non-white British ethnic background, sex chromosome aneuploidy (Data-Field 22019), genetic relatedness exclusions (kinship coefficient >0.0884, (7)), recommended genomic analysis exclusions (Data-Field 22010, poor heterozygosity/missingness), genetic and reported sex mismatch (5). After exclusion steps, a total of 3,283 LN cases and 277,506 controls were used for GWAS analysis. At the time of analysis (May 2022), we removed also participant who had withdrawn consent from the approved UKB project.

The UKB genetic data were imputed from genotype using the Haplotype Reference Consortium, UK10K and 1000 Genomes Project Phase 3 reference panels by IMPUTE4 software and resulted in nearly 96 million genetic variants (6). REGENIE v3.2 (8) was used for association analyses via a two-step procedure. REGENIE is a machine learning method for whole-genome regression that uses a two-step approach. The first step fits a whole genome regression model for individual trait predictions based on genetic data using the leave-one-chromosome-out (LOCO) scheme. Original non-imputed genotype data is used, filtering only high-quality genotyped variants: minor allele frequency (MAF) > 1%, minor allele count (MAC) > 5, genotyping rate >99%, Hardy-Weinberg equilibrium (HWE) test P > 10^−8^, <1% missingness. The quality control of genotype data and filtering was done by using plink2 software (9). Traits where the step 1 regression failed to converge due to case-control imbalances were subsequently excluded from subsequent analyses. The linear predictors from a logistic regression model using the LOCO method were saved and used as a fixed input value when fitting logistic regression models to test for association in step 2, which performs variant association analyses using the logistic whole-genome ridge-regression model with approximate saddle-point approximation (SPA) corrections when the p value from the standard logistic regression score test is below 0.01. Standard errors were computed from the effect size estimate and the likelihood ratio test P value. The association models in both steps also included the following covariates: age (cases: age at diagnosis, controls: age at recruitment), sex, genotyping array, and the first 10 precomputed genetic principal components (PCs, Data-Field 22009) (6).

After conducting GWAS with REGENIE, post-GWAS QC filters were applied to GWAS summary statistics for each subtype and phenocluster. Variants were removed for further analysis if they failed SPA corrections, MAF < 0.001 (in whole cohort) and imputation score < 0.9.

# **4. FinnGen**

The FinnGen study is a nationwide research initiative integrating genomic data with longitudinal health records from the Finnish population, aimed at advancing precision medicine (10). In its twelfth data release (R12), FinnGen includes genotype and phenotype data for over 500,000 individuals. Participants were genotyped using Illumina and Affymetrix arrays, and the resulting genotype data were imputed using a high-quality, population-specific reference panel built from over 8,000 whole-genome sequences from Finnish individuals. This approach ensured accurate imputation in a relatively homogeneous genetic population.

Phenotypic data in FinnGen were harmonized across multiple national registries, including hospital discharge records, cancer registries, procedure codes (NOMESCO), drug reimbursement codes from the Social Insurance Institution of Finland (KELA), and ATC-coded medication records. Disease phenotypes were defined using a combination of ICD-8, ICD-9, ICD-10, and ICD-O-3 codes. The complete list of phenotypes and their definitions is available on the FinnGen Risteys portal (https://r12.risteys.finregistry.fi/), which provides metadata including sample sizes, case definitions, and longitudinal information for each phenotype.

For this study, we selected lymphoid neoplasm subtypes with at least 100 cases to ensure adequate statistical power. In total, we identified ten individual LN subtypes that met this threshold. Additionally, FinnGen had already conducted joint GWAS analyses for broader composite endpoints, including the LN phenotype and the MM-MGUS phenocluster, which we incorporated directly into our meta-analyses. These phenotypes were harmonized with UK Biobank definitions to enable consistent cross-cohort analysis. Notably, FinnGen’s primary lymphoid malignancy endpoint (R12 pheno code CD2_PRIMARY_LYMPHOID_HEMATOPOIETIC_EXALLC) does not include MGUS. To address this, we performed a meta-analysis combining FinnGen’s LN summary statistics with MGUS to ensure consistency with the composite LN phenotype used in UKB and MVP. Detailed ICD codes, case and control count for each phenotype are provided in Supplementary Table 2. GWAS in FinnGen were conducted using REGENIE version 2.2.4 or 3.3, depending on the data freeze.

# **5. Million Veteran Program (MVP)**

The Million Veteran Program (MVP) is a national research initiative launched in 2011 by the U.S. Department of Veterans Affairs to investigate the genetic and environmental determinants of health and disease among U.S. military veterans (1). To date, MVP has enrolled over 900,000 participants, collecting biospecimens, genetic data, detailed survey information, and longitudinal electronic health records (EHRs), which include ICD-coded diagnoses, medications, laboratory values, and clinical procedures. These rich data allow for deep phenotyping and large-scale genomic analyses across a wide range of diseases, including rare cancers such as lymphoid neoplasms (LNs).

Participants were genotyped using a custom Affymetrix Axiom biobank array (MVP 1.0), which includes over 730,000 variants. Quality control steps included the exclusion of duplicate samples, individuals with excess heterozygosity or low call rates, and variants with high missingness, monomorphism, or deviation from Hardy–Weinberg equilibrium. Imputation was conducted using SHAPEIT4 and Minimac4 with reference to the 1000 Genomes Project Phase 3 and African Genome Resources panels. Ancestry assignment was performed using both the Harmonized Ancestry and Race/Ethnicity (HARE) method and genetically inferred ancestry (GIA), with the GIA-European (EUR) results used for the current analysis as they reflect the most updated and harmonized classification for population structure (11).

We used summary statistics generated by the MVP gwPheWAS initiative, specifically from the GIA-European ancestry subset. These results were derived using a GPU-accelerated version of SAIGE (SAIGE-GPU), which applies generalized linear mixed models to account for sample relatedness and case-control imbalance (12). Genetic association models were adjusted for age, sex, and the first ten principal components of ancestry. Post-GWAS quality control included exclusion of variants with poor imputation (r² < 0.3), minor allele count < 20, missing or implausible summary statistics, or extreme allele frequencies. Summary statistics were filtered using a modified EasyQC-based pipeline (13).

For this study, we included four LN-related single-subtype phenotypes: chronic lymphocytic leukemia (CLL; Phe_204_12), Hodgkin's lymphoma (HL; Phe_201), monoclonal gammopathy of undetermined significance (MGUS; Phe_270_32), and multiple myeloma (MM; Phe_204_4). The MGUS phenotype (Phe_270_32) includes both D47.2 (MGUS) and D89.1 (cryoglobulinemia); however, D89.1 is extremely rare in the population. For instance, only 38 cases were observed in UK Biobank in over 500.000 individuals. Therefore, the contribution of D89.1 to this composite phenotype is considered negligible, and we treated this phecode as representative of MGUS in all analyses.

In addition to individual subtypes, we incorporated two composite phenotypes: “cancer of other lymphoid and histiocytic tissue” (Phe_202) and “leukemia” (Phe_204). These broader definitions provided statistical power for multi-trait meta-analyses and included a wide range of lymphoid malignancies as defined by extensive ICD-10-CM mappings. To avoid overlapping case definitions, we meta-analyzed non-overlapping Phecodes—specifically Phe_201, Phe_202, Phe_204, and Phe_270_32—to create a harmonized LN summary statistic within MVP (Supplementary Figure 5).

These phenotype definitions and mappings were aligned with those used in UK Biobank and FinnGen to facilitate cross-cohort integration. Full details of MVP phenotype definitions, sample sizes, ICD-10-CM mappings, and links to the Phenomics VA portal are provided in Supplementary Table 3.


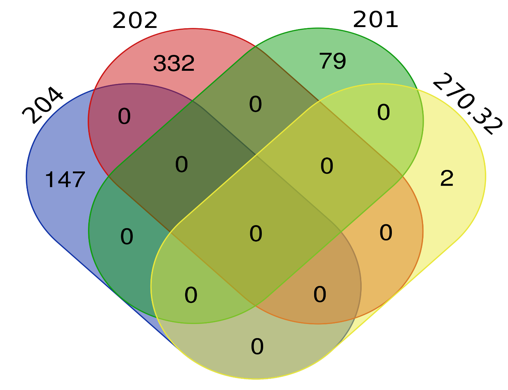


**Supplementary Figure 5.** The overlap between Phe201, Phe_202, Phe204 and Phe270.32 codes, number showing all ICD codes for given phenotype.

# **6. Replication in All of Us (AoU) and PLCO cohorts**

To replicate associations identified in the discovery meta-analysis, we used data from two independent cohorts: the All of Us (AoU) Research Program and the Prostate, Lung, Colorectal, and Ovarian (PLCO) Cancer Screening Trial. These cohorts provided phenotype-level summary statistics for selected lymphoid neoplasm (LN) subtypes and phenoclusters.

In AoU, four LN-related phenotypes were included: MGUS, MM, CLL, and a broader LN category. Phenotypes were defined using phecodeX identifiers and corresponding ICD-9-CM and ICD-10-CM codes. MGUS (BI_180.31) included ICD-9-CM code 273.1 and ICD-10-CM code D47.2. MM (CA_123.1) was defined by ICD-9-CM codes 203.0–203.02 and ICD-10-CM codes C90.0–C90.02. CLL (CA_121.21) was identified using ICD-9-CM codes 204.1–204.12 and ICD-10-CM codes C91.1, C91.10–C91.12, and C91.6, C91.60–C91.62. The composite LN phenotype (CA_120.2) included ICD-9-CM codes 200, 201, 202, and 204 and ICD-10-CM codes C81 through C91. Sample sizes ranged from 218 cases for MM to 1,072 for the composite LN phenotype, with control counts between 98,204 and 99,241.

In the PLCO cohort, summary statistics were available for CLL and the composite LN phenotype. While ICD-9 and ICD-10 codes were not reported, definitions were based on ICD-O-2 morphology codes. CLL was mapped using codes 9823 and 9670, while the broader LN phenotype was defined using a wide set of ICD-O-2 morphology codes: 9590–9595, 9650, 9652–9655, 9657–9667, 9663–9667, 9670–9677, 9680, 9684–9688, 9690–9698, 9700–9717, 9731, 9732, 9760–9764, 9820–9828, 9830, 9850, 9940, and 9941. Additionally, codes 9681–9683 were included when the topography code was not C499. In total, PLCO contributed 421 CLL cases and 1,820 LN cases, with corresponding control counts of 65,142 and 67,500, respectively.

These replication datasets were used to evaluate the consistency and robustness of novel loci identified in the primary meta-analysis across independent and ancestrally matched populations. All relevant case definition and numbers were given in the Supplementary Table 4.

# **7. Meta-analysis of individual subtypes**

We conducted genome-wide meta-analyses for eight individual LN subtypes across three cohorts: the UKB, FinnGen, and the MVP. Meta-analyses were performed using METAL (14), applying an inverse-variance weighted fixed-effects model. Subtypes were selected for inclusion if they had at least 100 cases in one or more of the cohorts.

Detailed case and control numbers for each subtype and cohort are provided in Supplementary Table 4.

To visualize the genome-wide results and assess inflation, we generated Manhattan and quantile–quantile (QQ) plots for each individual LN subtype GWAS (Supplementary Figures 6–13). The genomic inflation factors (λ_GC_) are reported in Supplementary Table 7.


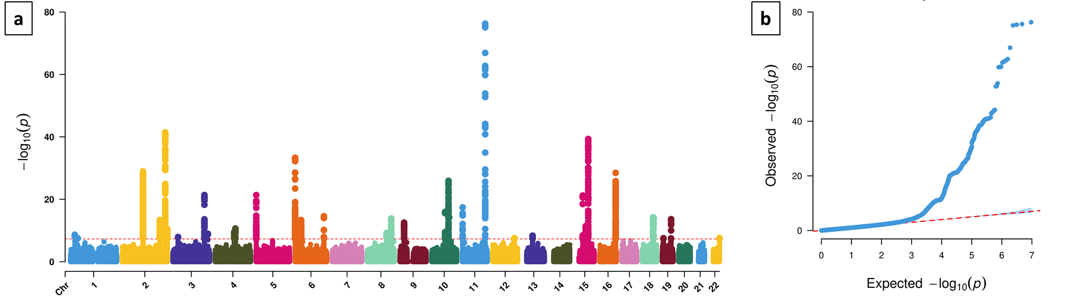


**Supplementary Figure 6.** Manhattan and QQ plots for the CLL GWAS. a) Manhattan plot x-axis chromosome numbers and y-axis negative log_10_P, b) Q-Q plot x and y axis show expected and observed negative log_10_P.


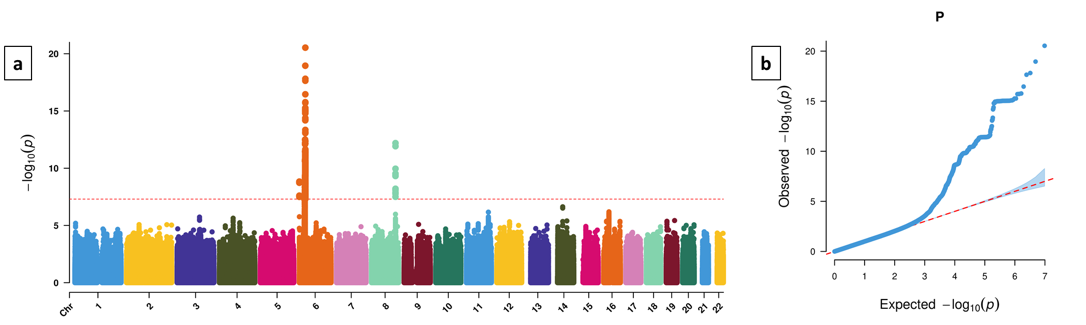


**Supplementary Figure 7.** Manhattan and QQ plots for the DLBCL GWAS. a) Manhattan plot x-axis chromosome numbers and y-axis negative log_10_P, b) Q-Q plot x and y axis show expected and observed negative log_10_P.


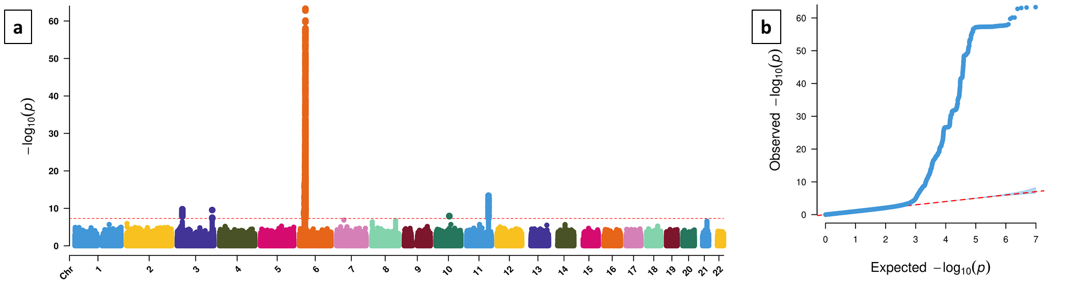


**Supplementary Figure 8.** Manhattan and QQ plots for the FL GWAS. a) Manhattan plot x-axis chromosome numbers and y-axis negative log_10_P, b) Q-Q plot x and y axis show expected and observed negative log_10_P.


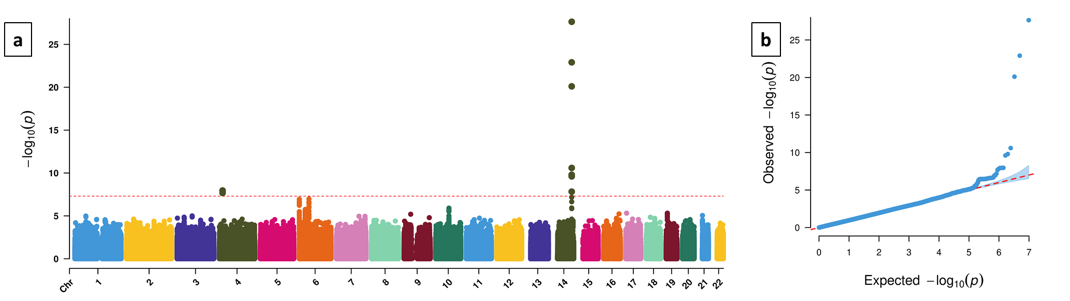


**Supplementary Figure 9.** Manhattan and QQ plots for the LPL-WM GWAS. a) Manhattan plot x-axis chromosome numbers and y-axis negative log_10_P, b) Q-Q plot x and y axis show expected and observed negative log_10_P.


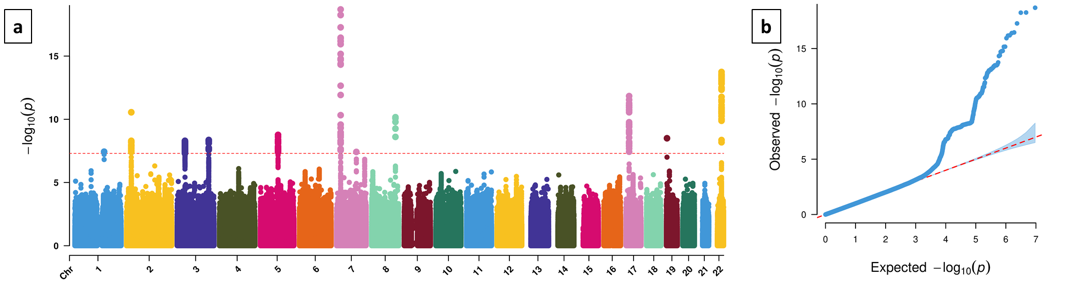


**Supplementary Figure 10.** Manhattan and QQ plots for the MM GWAS. a) Manhattan plot x-axis chromosome numbers and y-axis negative log_10_P, b) Q-Q plot x and y axis show expected and observed negative log_10_P.


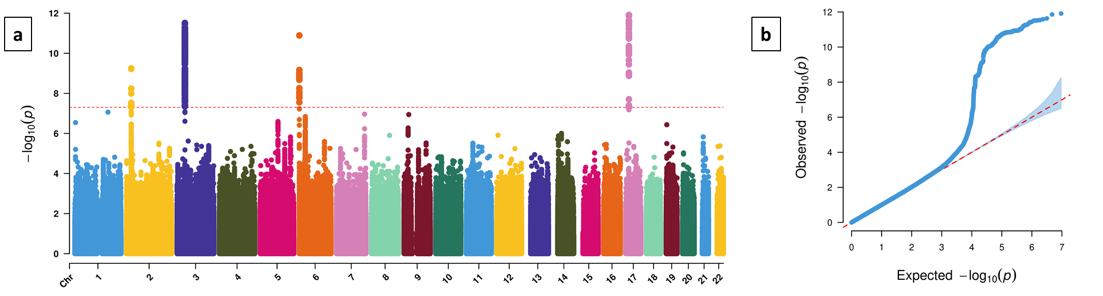


**Supplementary Figure 11.** Manhattan and QQ plots for the MGUS GWAS. a) Manhattan plot x-axis chromosome numbers and y-axis negative log_10_P, b) Q-Q plot x and y axis show expected and observed negative log_10_P.


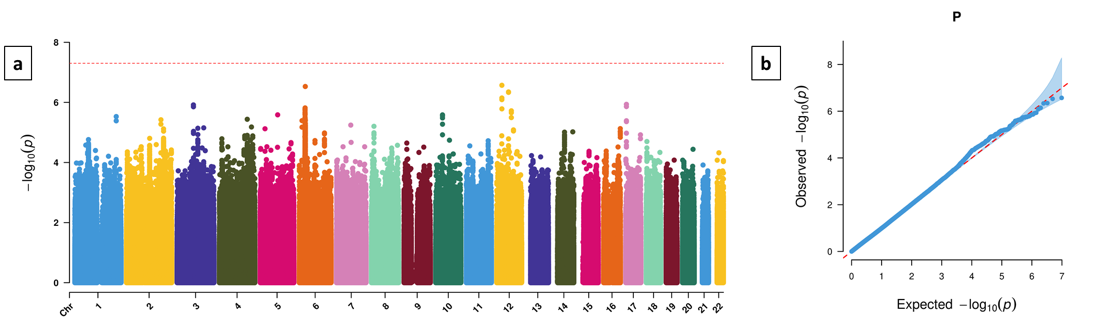


**Supplementary Figure 12.** Manhattan and QQ plots for the MZL GWAS. a) Manhattan plot x-axis chromosome numbers and y-axis negative log_10_P, b) Q-Q plot x and y axis show expected and observed negative log_10_P.


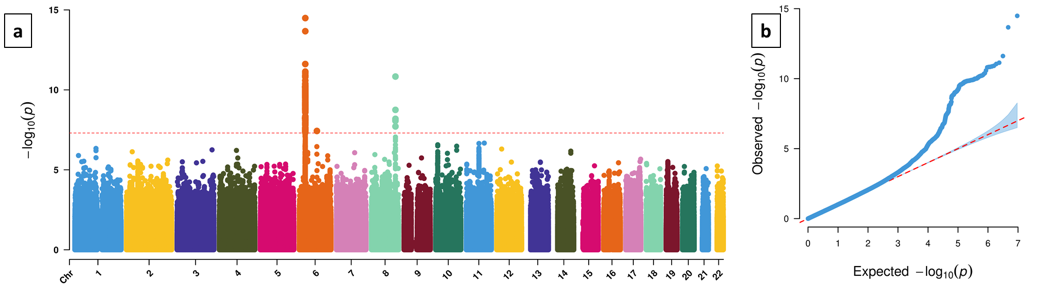


**Supplementary Figure 13.** Manhattan and QQ plots for the HL GWAS. a) Manhattan plot x-axis chromosome numbers and y-axis negative log_10_P, b) Q-Q plot x and y axis show expected and observed negative log_10_P.

# **8. Meta-analysis of phenoclusters**

We conducted genome-wide association meta-analyses for seven phenoclusters defined using hierarchical clustering of LN subtypes based on three independent biological dimensions: cell of origin (Cell-P and Cell-B), somatic mutation profiles (Soma-G1 and Soma-G2), and approved drug usage profiles (Drug-G1). In addition, we analyzed a composite phenotype for MGUS and MM, as well as a broad LN phenotype that included all LN subtypes with sufficient case numbers across cohorts. Meta-analyses were performed using METAL (14) with an inverse-variance weighted fixed-effects model.

Detailed sample counts, subtype compositions, and phenotype definitions used in each cohort are provided in Supplementary Table 4.

Manhattan and QQ plots were generated for each phenocluster GWAS (Supplementary Figures 14–20). The λ_GC_ values are summarized in Supplementary Table 7.


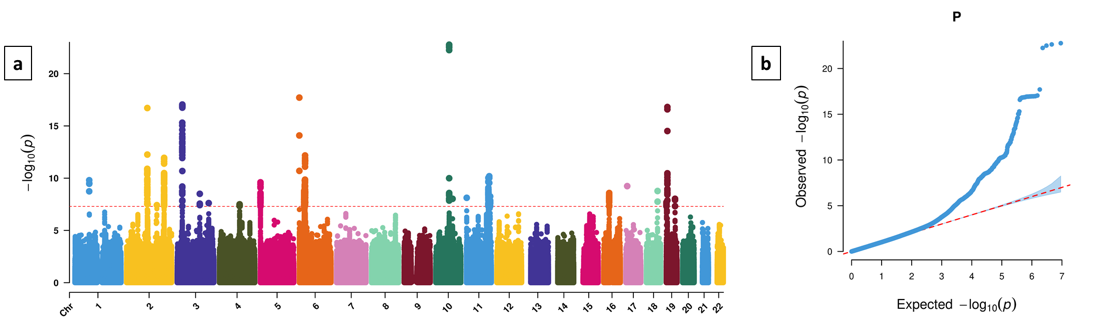


**Supplementary Figure 14.** Manhattan and QQ plots for the Cell-B GWAS. a) Manhattan plot x-axis chromosome numbers and y-axis negative log_10_P, b) Q-Q plot x and y axis show expected and observed negative log_10_P.


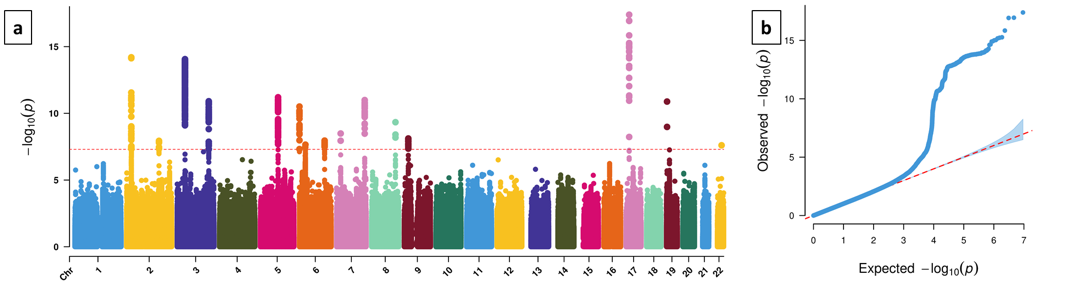


**Supplementary Figure 15.** Manhattan and QQ plots for the Cell-P GWAS. a) Manhattan plot x-axis chromosome numbers and y-axis negative log_10_P, b) Q-Q plot x and y axis show expected and observed negative log_10_P.


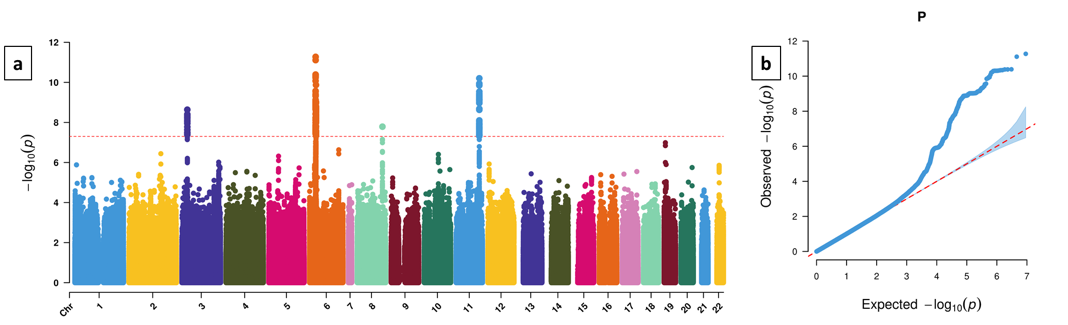


**Supplementary Figure 16.** Manhattan and QQ plots for the Soma-G1 GWAS. a) Manhattan plot x-axis chromosome numbers and y-axis negative log_10_P, b) Q-Q plot x and y axis show expected and observed negative log_10_P.


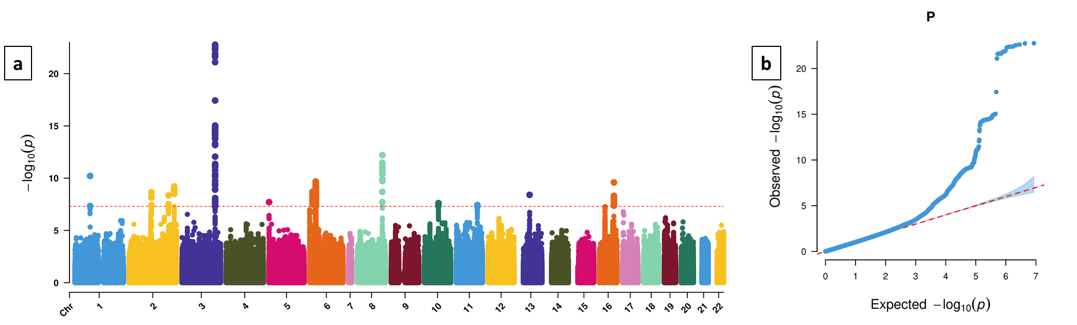


**Supplementary Figure 17.** Manhattan and QQ plots for the Soma-G2 GWAS. a) Manhattan plot x-axis chromosome numbers and y-axis negative log_10_P, b) Q-Q plot x and y axis show expected and observed negative log_10_P.


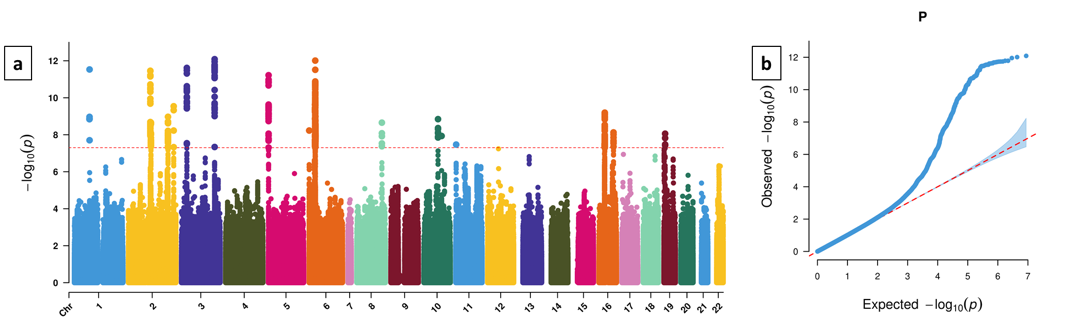


**Supplementary Figure 18.** Manhattan and QQ plots for the Drug-G1 GWAS. a) Manhattan plot x-axis chromosome numbers and y-axis negative log_10_P, b) Q-Q plot x and y axis show expected and observed negative log_10_P.


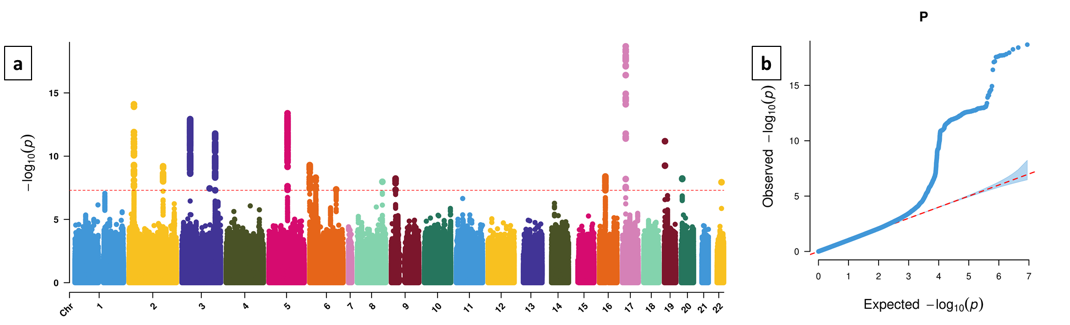


**Supplementary Figure 19.** Manhattan and QQ plots for the MM-MGUS GWAS. a) Manhattan plot x-axis chromosome numbers and y-axis negative log_10_P, b) Q-Q plot x and y axis show expected and observed negative log_10_P.


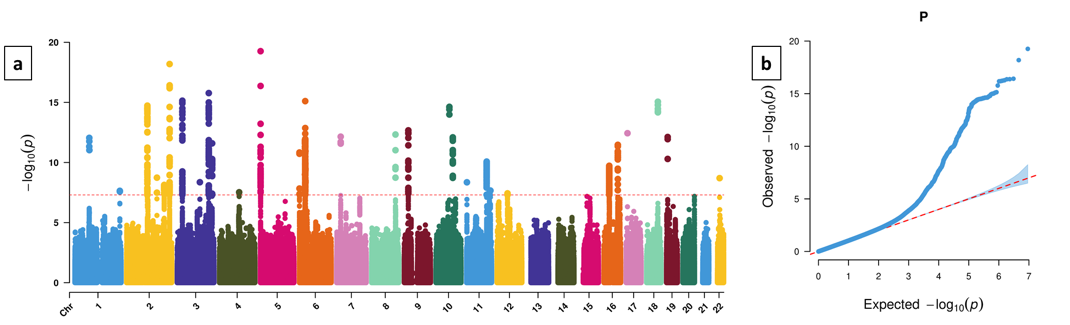


**Supplementary Figure 20.** Manhattan and QQ plots for the LN GWAS. a) Manhattan plot x-axis chromosome numbers and y-axis negative log_10_P, b) Q-Q plot x and y axis show expected and observed negative log_10_P.

# **9. ASSET**

As a hypothesis-free strategy to identify pleiotropic loci across LN subtypes, we applied the Association analysis based on SubSETs (ASSET) framework. ASSET (15) is designed to detect association signals that may be present only in a subset of traits and potentially in opposite directions. It systematically evaluates all possible non-empty subsets of traits for association with each variant, correcting for multiple testing while accounting for sample overlap and correlated test statistics.

We implemented ASSET using a custom R script optimized for parallel computation across phenotypes and genomic regions. Both one-sided and two-sided models were run. The one-sided analysis identifies subsets with consistent direction of effect (risk-increasing or risk-decreasing), while the two-sided model allows for directional heterogeneity, enabling detection of loci with opposite effects in different subtypes. We included ten LN subtypes in the ASSET analysis: CLL, FL, HL, DLBCL, MZL, MM, and LPL-WM. Sample sizes for each subtype were harmonized across cohorts (UKB, FinnGen, and MVP), as summarized in Supplementary Table 4. The total number of cases ranged from 361 (LPL-WM) to 5,106 (MGUS), with control sizes ranging from ~378,000 to over 1.2 million, depending on the cohort composition. The ASSET analyses enabled us to identify pleiotropic variants that might not be captured through single-trait or phenocluster-based approaches and provided an additional layer of insight into the shared genetic architecture across LN subtypes. Manhattan and QQ plots were generated for ASSET1-sided GWAS to evaluate the distribution of association signals and to assess test statistic inflation (Supplementary Figure 21).


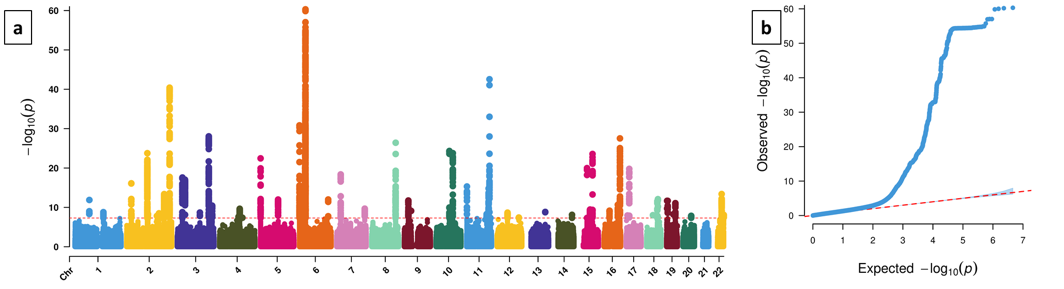


**Supplementary Figure 21.** Manhattan and QQ plots for the ASSET 1-sided analysis. a) Manhattan plot x-axis chromosome numbers and y-axis negative log_10_P, b) Q-Q plot x and y axis show expected and observed negative log_10_P.

To compare the captured association signals from ASSET1-sided analysis, phenoclusters and individual, we generated a multi-phenotype Miami plot which is visually indicate contributor subtypes and comparison between phenocluster approach and ASSET (Supplementary Figure 22).


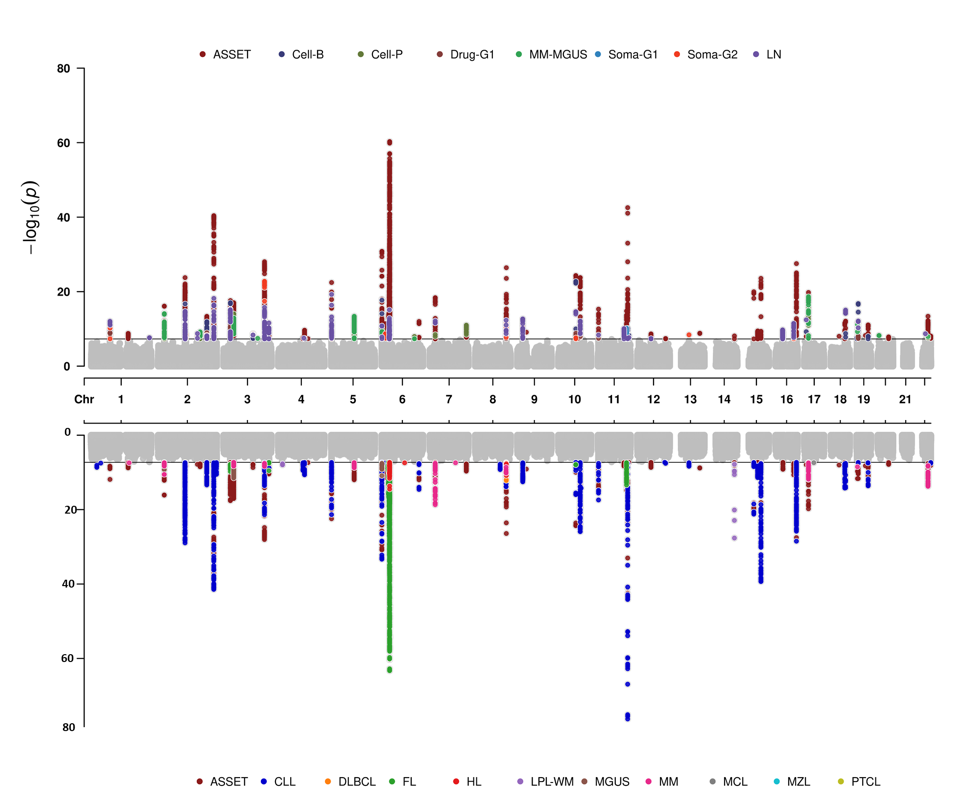


**Supplementary Figure 22.** The captured association signals from ASSET1-sided analysis, phenoclusters and individual subtypes. The signals from ASSET are shown in firebrick color. Individual subtypes and phenoclusters with distinct colors. X-axis shows chromosome numbers and y-axis negative log_10_P. The upside of the Miami plot is for ASSET versus phenocluster and the down side for ASSET versus individual subtypes.

# **10. Identification of driver and contributor subtypes from multi-trait**

While multi-trait GWAS approaches are increasingly applied to improve discovery power, most studies stop at identifying shared loci and do not dissect which individual traits or disease subtypes are driving the associations. In the context of LNs, this is particularly important given the heterogeneity of subtypes and the frequent co-occurrence of pleiotropic loci.

To systematically identify driver subtypes for each pleiotropic association, we integrated evidence from multiple sources. First, we used Hypothesis Prioritization in Multi-Trait Colocalization (HyPrColoc), a Bayesian method that identifies whether signals across traits share a common causal variant. Second, we leveraged single-subtype GWAS results to assess significance at each multi-trait locus. Third, we cross-referenced known loci previously reported for individual subtypes.

For each genome-wide significant locus identified through phenocluster-based or ASSET-based analyses, we assigned subtypes as primary or supportive contributors. Subtypes were labeled primary if they showed colocalization with high posterior probability (PP > 0.7) or reached genome-wide significance in individual GWAS; supportive contributors were defined based on suggestive significance (P < 1×10⁻⁶) or moderate colocalization evidence. All assessments are detailed in Supplementary Table 11.

# **11. LDSC**

To quantify the extent of shared genetic architecture among LN subtypes and phenoclusters, we performed genome-wide genetic correlation analyses using linkage disequilibrium score regression (LDSC). LDSC (16) estimates genetic correlation (rg) between traits based on GWAS summary statistics, accounting for linkage disequilibrium (LD) patterns across the genome.

We applied LDSC v1.0.1 using the munge_sumstats.py utility to format summary statistics for each of the ten LN subtypes and selected phenoclusters. Analyses were restricted to HapMap3 variants, following best practices to ensure reliable heritability and correlation estimates. We excluded variants with minor allele frequency (MAF) below 5% and all variants located in the extended major histocompatibility complex (MHC) region on chromosome 6 (25–35 Mb), due to its complex LD structure. The pre-computed LD scores for European ancestry were downloaded from the Alkes Group website (<https://console.cloud.google.com/storage/browser/broad-alkesgroup-public-requester-pays/>).

The effective sample sizes (N_eff_) were calculated with:

$[Neff=4/(1/Ncases+1/Ncontrols)]$

Bivariate genetic correlations were calculated for all pairwise combinations of LN subtypes and selected phenoclusters. Statistical significance was determined using a Bonferroni-corrected threshold of P ≤ 0.005, accounting for multiple testing. Results are reported in Supplementary Table 14 and visualized in Figure 3c of the main manuscript.

# **12. MAGMA set-based analysis**

To identify genes associated with LNs beyond individual variant-level signals, we performed gene-level association testing using MAGMA (Multi-marker Analysis of GenoMic Annotation). MAGMA aggregates SNP-level association statistics within genes, accounting for LD, and provides a robust approach for identifying genes with modest but consistent association signals.

We implemented MAGMA v1.08 as integrated within the FUMA platform (version 1.5.2), using GWAS meta-analysis summary statistics from both individual LN subtypes and phenocluster-level analyses. We used gene window parameter as 5 kb upstream and downstream of the genes. SNPs were mapped to 19,010 protein-coding genes based on NCBI 37.3 gene definitions. We applied a Bonferroni-corrected threshold of P < 2.63 × 10⁻⁶ to define genome-wide significance.

Genome-wide significant genes were identified for several subtypes and phenoclusters, including genes located outside of lead GWAS loci, supporting independent association signals. The full list of statistically significant genes across analyses is provided in Supplementary Table 17, with gene-locus mappings detailed in Supplementary Table 18.

These gene-level results were used as one component of our multi-layered locus-to-gene mapping framework, alongside fine-mapping, molecular QTL data, and machine learning–based prioritization (e.g., FLAMES and Open Targets). By combining SNP- and gene-level evidence, we improved resolution in identifying likely effector genes contributing to LN susceptibility.

# **13. Locus to gene mapping**

To prioritize candidate effector genes at genome-wide significant loci associated with LNs, we applied an integrative locus-to-gene mapping strategy. This approach combined statistical association, fine-mapped variant function, regulatory annotation, and gene-level enrichment to assign the most likely gene(s) at each locus.

We used four complementary methods to derive gene assignments:

1. **FLAMES (Fine-mapped Locus Assessment Model of Effector Signals)**: a machine learning–based framework that aggregates multiple genomic annotations, including chromatin state, variant consequence, and regulatory potential, to predict effector genes at fine-mapped loci. FLAMES combines a broad range of data sources, including pathogenicity scores (CADD and VEP), enhancer–promoter interactions derived from promoter capture Hi-C, and interaction modeling from ABC enhancer–promoter links, ABC CRISPR screens, and Cicero-predicted cis-regulatory interactions in whole blood. Additionally, it incorporates quantitative trait loci (QTL) data from GTEx, eQTLGen, and regulatory QTLs (rQTLs) for differential splicing and transcript usage. Gene-enhancer associations were further informed by datasets such as HACER CAGE, HACER GRO/PRO-seq, FANTOM5, EpiMap, GeneHancer, and the Roadmap Epigenomics Project. Gene-level associations from MAGMA (20), polygenic priority scores (PoPS) (21), and positional annotations (e.g., promoter overlap, distance to gene, weighted distance to transcription start site) were also included in the model. Fine-mapped credible sets were used as input to FLAMES, along with MAGMA gene-based association results. For seven loci where fine-mapping failed, we instead used the lead SNP and its high-LD proxies (R² ≥ 0.8, GWAS P < 1×10⁻⁵) as input variants for locus-to-gene mapping. Genes prioritized by FLAMES are provided in Supplementary Table 19.
2. **MAGMA Gene-Based Association**: genome-wide MAGMA results (Section 14) were used to identify genes showing significant enrichment of SNP-level signals across the gene body. Gene associations that passed the Bonferroni-corrected threshold are listed in Supplementary Tables 17–18.
3. **Open Targets Locus-to-Gene (L2G) Scores**: we leveraged scores from the Open Targets Genetics Platform, which integrates functional genomic data, variant proximity, and fine-mapped causal probabilities to assign the most likely gene(s) per locus (Supplementary Table 20).
4. **cis-Molecular QTL Mapping**: fine-mapped variants were intersected with expression QTL (eQTL), splicing QTL (sQTL), protein QTL (pQTL), and single-cell QTL datasets derived from blood and hematopoietic tissues. QTL-supported genes for each locus are listed in Supplementary Table 22.
5. **VEP**: In addition, we annotated fine-mapped variants using Ensembl Variant Effect Predictor (VEP, v113) to assess protein-altering consequences and regulatory element overlaps (Supplementary Table 21).

Each gene was evaluated across methods using a binary scoring system (1 if supported, 0 otherwise), and a composite score was calculated by averaging support across the four main lines of evidence. Genes supported by two or more independent approaches were considered high-confidence effector candidates. This final set of prioritized genes across all loci is presented in Supplementary Table 23, with a summary overview in Supplementary Table 24 and visualized in Figure 4 of the main manuscript.

This integrative framework enabled systematic identification of genes most likely to mediate the observed genetic associations, laying the foundation for downstream functional studies and therapeutic exploration.

# **14. Risk Gene and Enrichment Analysis**

To evaluate the biological relevance and translational potential of the prioritized genes identified through our locus-to-gene mapping framework, we conducted a series of enrichment analyses focused on gene function, tissue and cell-type specificity, and therapeutic tractability.

We first performed **Gene Ontology (GO) biological process enrichment analysis** using the STRING database (v12), applying an FDR threshold of <0.05. This analysis revealed that the full set of 131 prioritized genes (Supplementary Table 23) was significantly enriched in pathways related to immune regulation, lymphocyte activation, hematopoietic development, transcriptional control, and apoptosis (Supplementary Table 23). When focusing exclusively on the 78 genes mapped from **novel loci**, a similar but more refined pattern was observed, with strong enrichment for B-cell proliferation, RNA polymerase II–mediated transcription, and DNA damage response pathways (Supplementary Table 25).

We next assessed **cell-type specificity** using WebCSEA, which evaluates enrichment across over 1,300 cell types and tissues. The full gene set showed strong enrichment in immune cells—particularly B cells, plasma cells, T cells, and natural killer (NK) cells (Supplementary Table 26; Figure 5c). Genes derived from novel loci maintained this immune-focused pattern but showed more specific enrichment in naïve and memory B cells, suggesting subtype-specific immune contexts (Supplementary Table 26; Figure 5d).

To assess **druggability**, we queried the Drug–Gene Interaction Database (DGIdb) and the Open Targets Platform. A total of 1,258 drug–gene interaction pairs were identified, involving 453 drugs with Anatomical Therapeutic Chemical (ATC) classifications (Supplementary Table 28). ATC enrichment analysis (Supplementary Table 29, Supplementary Figure 23) revealed a significant overrepresentation of antineoplastic and immunomodulatory agents (ATC Level 1: L; OR = 9.49, P = 6.8×10⁻⁶³), particularly those used in hematologic malignancies (ATC Level 2: L01; OR = 13.8, P = 7.7×10⁻⁷¹). Additionally, 16 prioritized genes were identified as targets of drugs either approved or in clinical trials, further supporting their translational potential (Supplementary Table 30; Table 3 in the main manuscript).


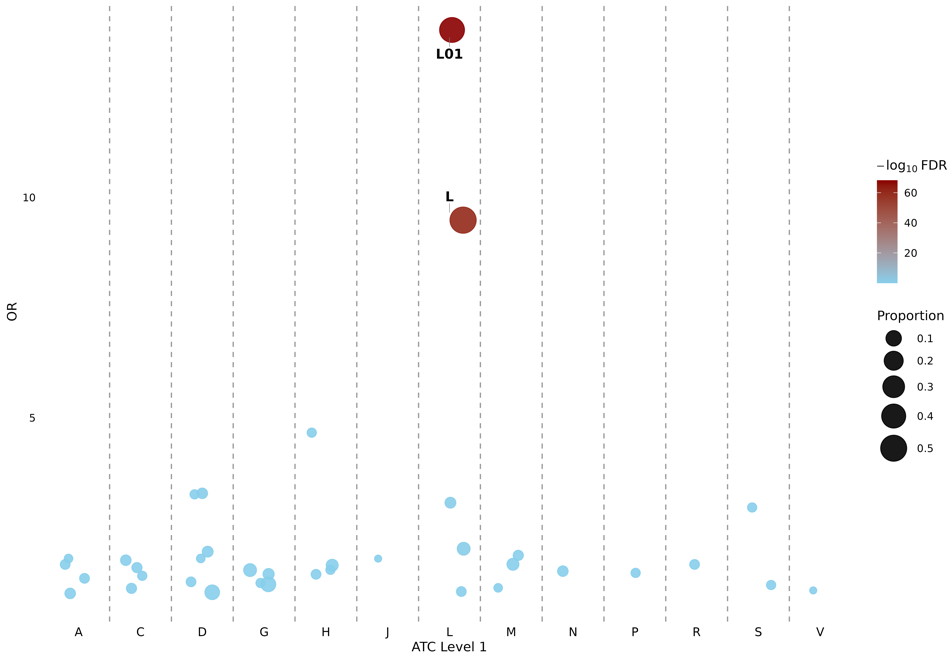


**Supplementary Figure 23.** The enrichment analysis results ATC code level 1 and 2 for DGI identified drugs. As background all DrugBank ATC codes are were taken. The x axis shows level 1 categories and y axis show odd ratio. The size of points is proportion of given category in the drug set and blue to red color shows negative log_10_FDR.

Notably, our drug–gene interaction results also uncovered links between risk genes and **environmental exposures.** For example, PAX5—prioritized at a novel pleiotropic locus shared between CLL and MGUS—was found to interact with 2,3,7,8-tetrachlorodibenzo-p-dioxin (TCDD), a known immunotoxicant and the primary toxic component of Agent Orange. This highlights the potential interplay between inherited genetic risk and environmental triggers in LN pathogenesis.

Together, these findings demonstrate that the genes identified in our study are not only biologically coherent—functionally and anatomically—but also relevant for therapeutic targeting. Our enrichment analyses reinforce the potential of using multi-trait genetic discovery to uncover both disease biology and clinical opportunities in lymphoid neoplasms.

# **Supplementary References**

1. R Core Team. R: A Language and Environment for Statistical Computing [Internet]. Vienna, Austria; 2023. Available from: https://www.R-project.org/

2. Galili T. dendextend: an R package for visualizing, adjusting and comparing trees of hierarchical clustering. Bioinformatics [Internet]. 2015 Nov 15;31(22):3718–20. Available from: http://dx.doi.org/10.1093/bioinformatics/btv428

3. Warnes G, Bolker B, Bonebakker L, Gentleman R, Liaw W, Lumley T, et al. Gplots: Various R programming tools for plotting data [Internet]. 2015. Available from: https://github.com/talgalili/gplots

4. Fowlkes EB, Mallows CL. A method for comparing two hierarchical clusterings. J Am Stat Assoc [Internet]. 1983 Sep;78(383):553. Available from: http://dx.doi.org/10.2307/2288117

5. Sudlow C, Gallacher J, Allen N, Beral V, Burton P, Danesh J, et al. UK biobank: An open access resource for identifying the causes of a wide range of complex diseases of middle and old age. PLoS Med [Internet]. 2015 Mar 31;12(3):e1001779. Available from: http://dx.doi.org/10.1371/journal.pmed.1001779

6. Bycroft C, Freeman C, Petkova D, Band G, Elliott LT, Sharp K, et al. The UK Biobank resource with deep phenotyping and genomic data. Nature [Internet]. 2018 Oct;562(7726):203–9. Available from: http://dx.doi.org/10.1038/s41586-018-0579-z

7. Manichaikul A, Mychaleckyj JC, Rich SS, Daly K, Sale M, Chen W-M. Robust relationship inference in genome-wide association studies. Bioinformatics [Internet]. 2010 Nov 15;26(22):2867–73. Available from: http://dx.doi.org/10.1093/bioinformatics/btq559

8. Mbatchou J, Barnard L, Backman J, Marcketta A, Kosmicki JA, Ziyatdinov A, et al. Computationally efficient whole-genome regression for quantitative and binary traits. Nat Genet [Internet]. 2021 Jul;53(7):1097–103. Available from: http://dx.doi.org/10.1038/s41588-021-00870-7

9. Chang CC, Chow CC, Tellier LC, Vattikuti S, Purcell SM, Lee JJ. Second-generation PLINK: rising to the challenge of larger and richer datasets. Gigascience [Internet]. 2015 Dec;4(1). Available from: http://dx.doi.org/10.1186/s13742-015-0047-8

10. Kurki MI, Karjalainen J, Palta P, Sipilä TP, Kristiansson K, Donner KM, et al. FinnGen provides genetic insights from a well-phenotyped isolated population. Nature [Internet]. 2023 Jan;613(7944):508–18. Available from: http://dx.doi.org/10.1038/s41586-022-05473-8

11. Fang H, Hui Q, Lynch J, Honerlaw J, Assimes TL, Huang J, et al. Harmonizing genetic ancestry and self-identified race/ethnicity in genome-wide association studies. Am J Hum Genet [Internet]. 2019 Oct 3;105(4):763–72. Available from: http://dx.doi.org/10.1016/j.ajhg.2019.08.012

12. Zhou W, Nielsen JB, Fritsche LG, Dey R, Gabrielsen ME, Wolford BN, et al. Efficiently controlling for case-control imbalance and sample relatedness in large-scale genetic association studies. Nat Genet [Internet]. 2018 Sep;50(9):1335–41. Available from: http://dx.doi.org/10.1038/s41588-018-0184-y

13. Winkler TW, Day FR, Croteau-Chonka DC, Wood AR, Locke AE, Mägi R, et al. Quality control and conduct of genome-wide association meta-analyses. Nat Protoc [Internet]. 2014 May;9(5):1192–212. Available from: http://dx.doi.org/10.1038/nprot.2014.071

14. Willer CJ, Li Y, Abecasis GR. METAL: fast and efficient meta-analysis of genomewide association scans. Bioinformatics [Internet]. 2010 Sep 1;26(17):2190–1. Available from: http://dx.doi.org/10.1093/bioinformatics/btq340

15. Bhattacharjee S, Rajaraman P, Jacobs KB, Wheeler WA, Melin BS, Hartge P, et al. A subset-based approach improves power and interpretation for the combined analysis of genetic association studies of heterogeneous traits. Am J Hum Genet [Internet]. 2012 May 4;90(5):821–35. Available from: http://dx.doi.org/10.1016/j.ajhg.2012.03.015

16. Bulik-Sullivan BK, Schizophrenia Working Group of the Psychiatric Genomics Consortium, Loh P-R, Finucane HK, Ripke S, Yang J, et al. LD Score regression distinguishes confounding from polygenicity in genome-wide association studies. Nat Genet [Internet]. 2015 Mar;47(3):291–5. Available from: http://dx.doi.org/10.1038/ng.3211
